# Supplementary material for: Robust normalization and transformation techniques for constructing gene coexpression networks from RNA-seq data
Source: Genome Biol. 2022 Jan 3;23:1. doi: 10.1186/s13059-021-02568-9 (PMC8721966; doi:10.1186/s13059-021-02568-9)
Supplement: Supplementary file 1 — Additional file 1: Supplemental Figures and a Note. This file contains supplemental figures S1-S16 and a Supplemental Note describing various aspects of the gold standard. [file 13059_2021_2568_MOESM1_ESM.pdf]

Additional file 1: Supplemental Figures and a Note.

This file contains supplemental figures S1-S16 and a Supplemental Note describing various aspects of the gold standard.

**Figure S1**. Recount2 data used in this study.	2
**Figure S2**. Overall performance of workflows based on the tissue-aware gold standard.	3
**Figure S3**. Dataset-level pairwise comparison of workflow performance for SRA datasets based on the tissue-naive gold standard.	4
**Figure S4**. Dataset-level pairwise comparison of workflow performance for GTEx and SRA datasets based on the tissue-aware gold standard.	5
**Figure S5**. Dataset-level pairwise comparison of workflow performance for SRA datasets based on the tissue-aware gold standard.	6
**Figure S6**. Impact of individual methods on performance of workflows based on the tissue-aware gold standard.	7
**Figure S7**. Impact of various dataset-related experimental factors on performance of workflows based on the tissue-aware gold standard.	8
**Figure S8**. Overall performance of workflows and pairwise-comparison using refine.bio datasets based on the tissue-aware gold standard.	10
**Figure S9**. Overlap between the tissue-aware gold standards.	11
**Figure S10**. Overall accuracy of coexpression networks when evaluated based on the tissue-naive and tissue-aware gold standards.	12
**Figure S11**. Overall performance of top workflows with and without asinh transformation based on the tissue-naive gold standard.	13
**Figure S12**. Performance of workflows utilizing different data transformation methods based on the tissue-naive gold standard.	14
**Figure S13**. Performance of workflows utilizing different data transformation methods based on the tissue-aware gold standard.	15
**Figure S14**. Overall performance of workflows based on the tissue-naive gold standard.	16
**Figure S15**. Overall performance of workflows based on the tissue-aware gold standard.	17
**Figure S16**. Overall performance of top ten workflows using Pearson and Spearman correlation based on the tissue-naive gold standard.	18
**Supplemental Note**	19

Supplemental Figures

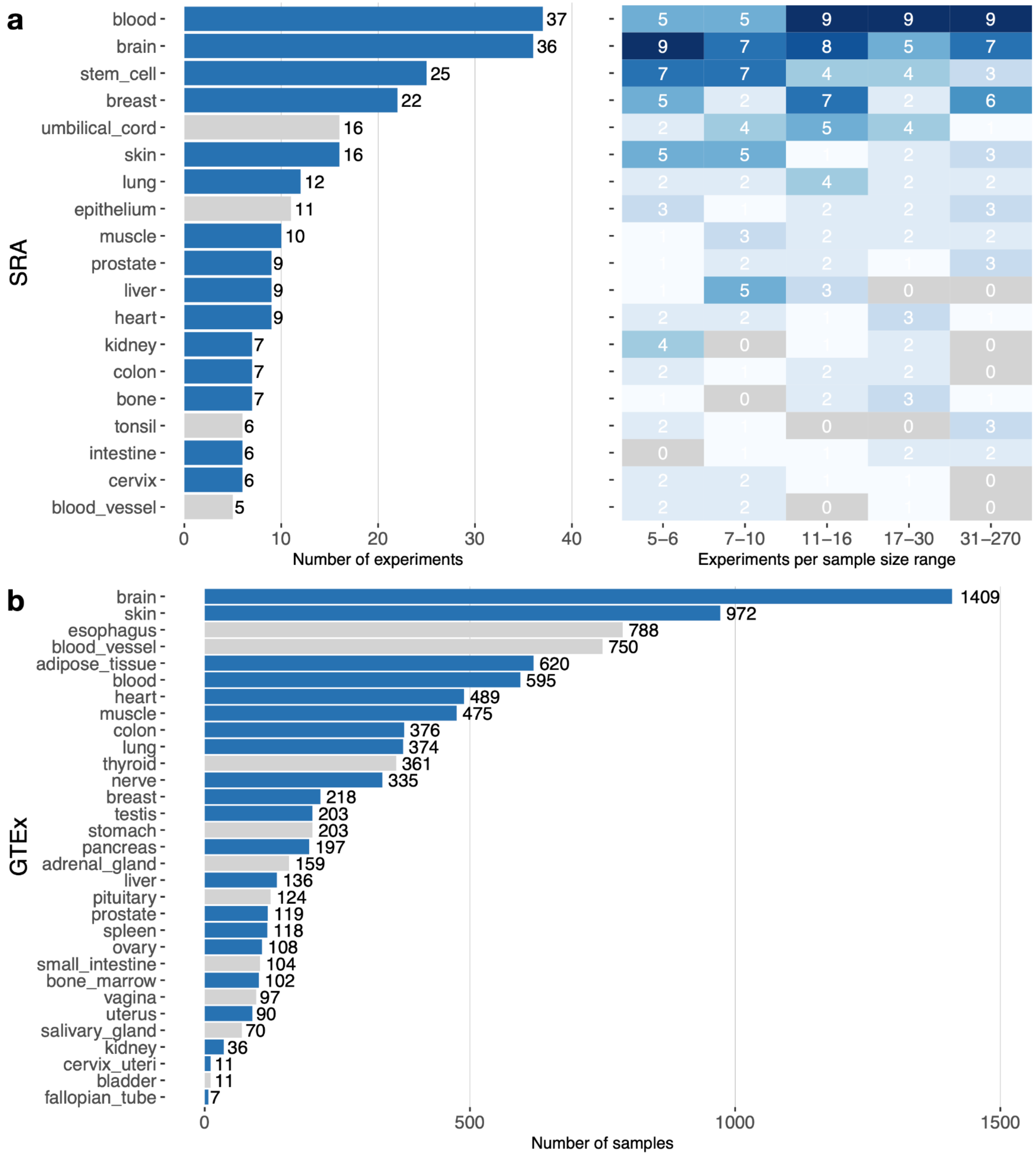

Figure S1. Recount2 data used in this study. (**a**) The barplot shows the number of experiments from each tissue in the SRA data. The heatmap on the right shows the number of projects/experiments that have a particular sample size for each tissue. (**b**) The barplot shows the number of samples for each GTEx tissue. In the barplots, blue bars indicate tissues for which we were able to create a tissue-aware gold standard. Tissues with gray bars were evaluated on the tissue-naive standard only.

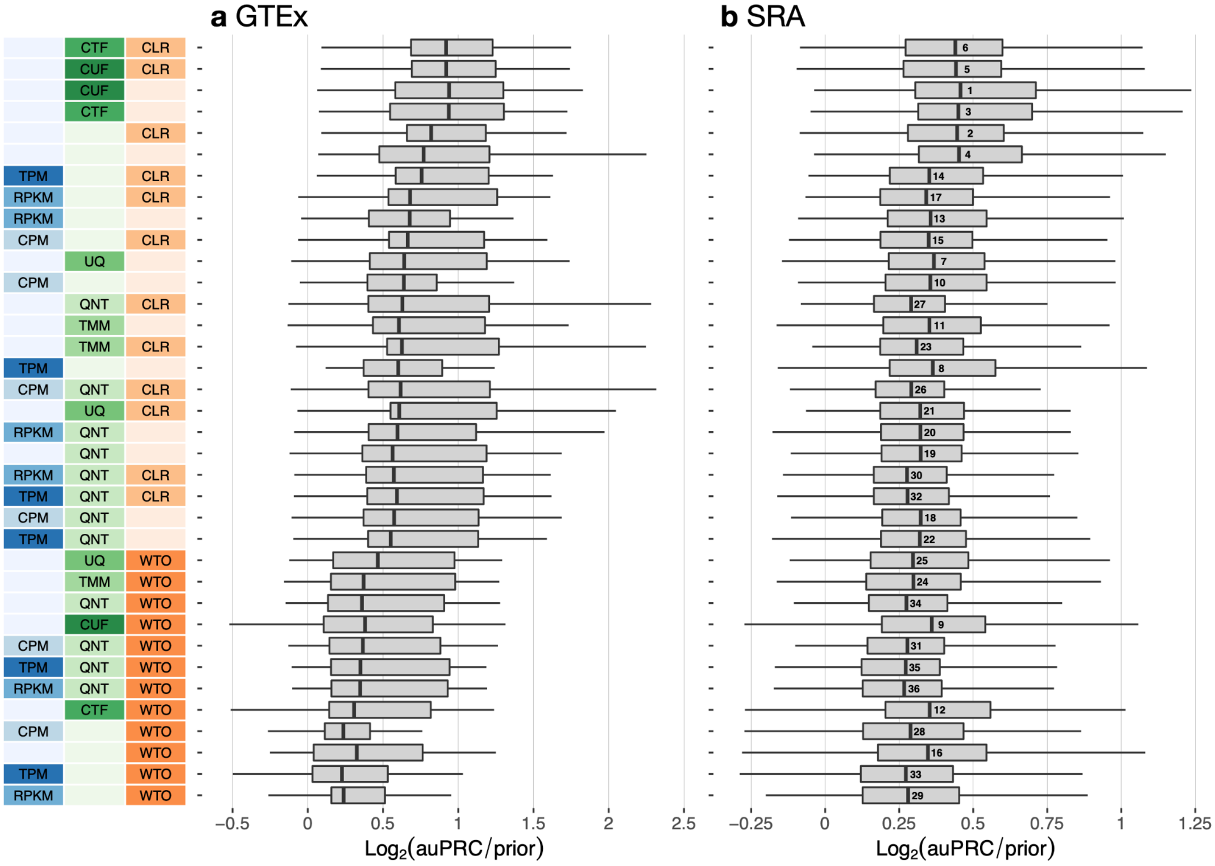

Figure S2. Overall performance of workflows based on the tissue-aware gold standard. The plots show the aggregate accuracy of all coexpression networks resulting from each individual workflow using (**a**) GTEx and (**b**) SRA datasets, evaluated based on the tissue-aware gold standard. The workflows (rows) are described in terms of the specific method used in the within-sample normalization (blues), between-sample normalization (greens), and network transformation (oranges) stages. The performance of each workflow is presented as boxplots (without outliers) that summarizes the log2(auPRC/prior) of each workflow where auPRC is the area under the precision recall curve (see Methods). The workflows are ordered by their median log2(auPRC/prior) for the GTEx data. The numbers inside the SRA boxes indicate rank by median log2(auPRC/prior) of the workflows for the SRA data. *Figure 2* contains these performance plots based on the tissue-naive gold standard.

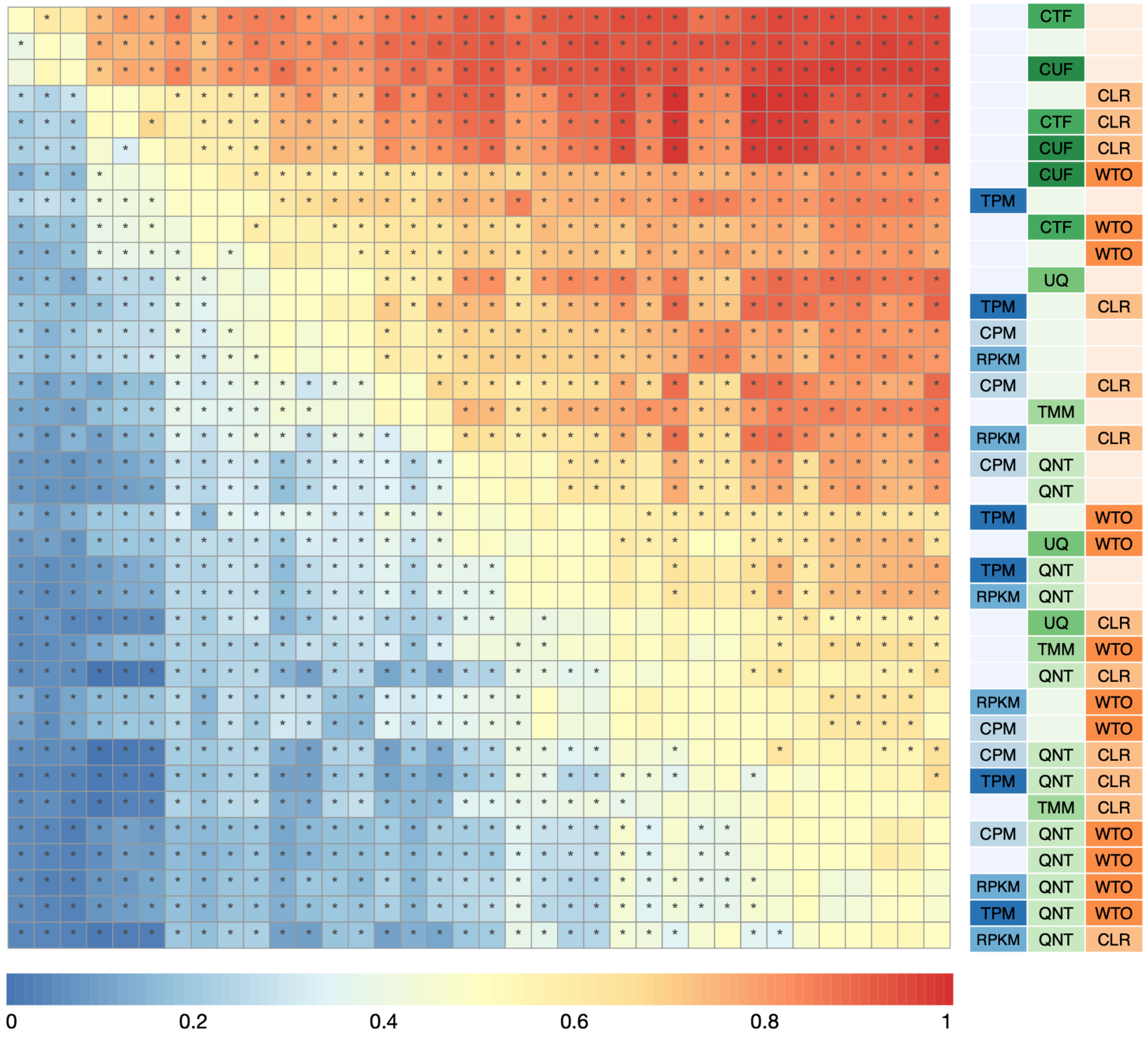

Figure S3. Dataset-level pairwise comparison of workflow performance for SRA datasets based on the tissue-naive gold standard. The heatmap shows the relative performance of a pair of workflows, corresponding to a row and a column, directly compared to each other for the SRA datasets based on the tissue-naive gold standard. The color in each cell (row, column) represents the proportion of datasets for which the workflow along the row has a higher log2(auPRC/prior) than the workflow along the column. Comparisons that are statistically significant (corrected p < 0.01) based on a paired Wilcoxon test are marked with an asterisk. *Figures 3a* contains the corresponding heatmap for GTEx datasets.

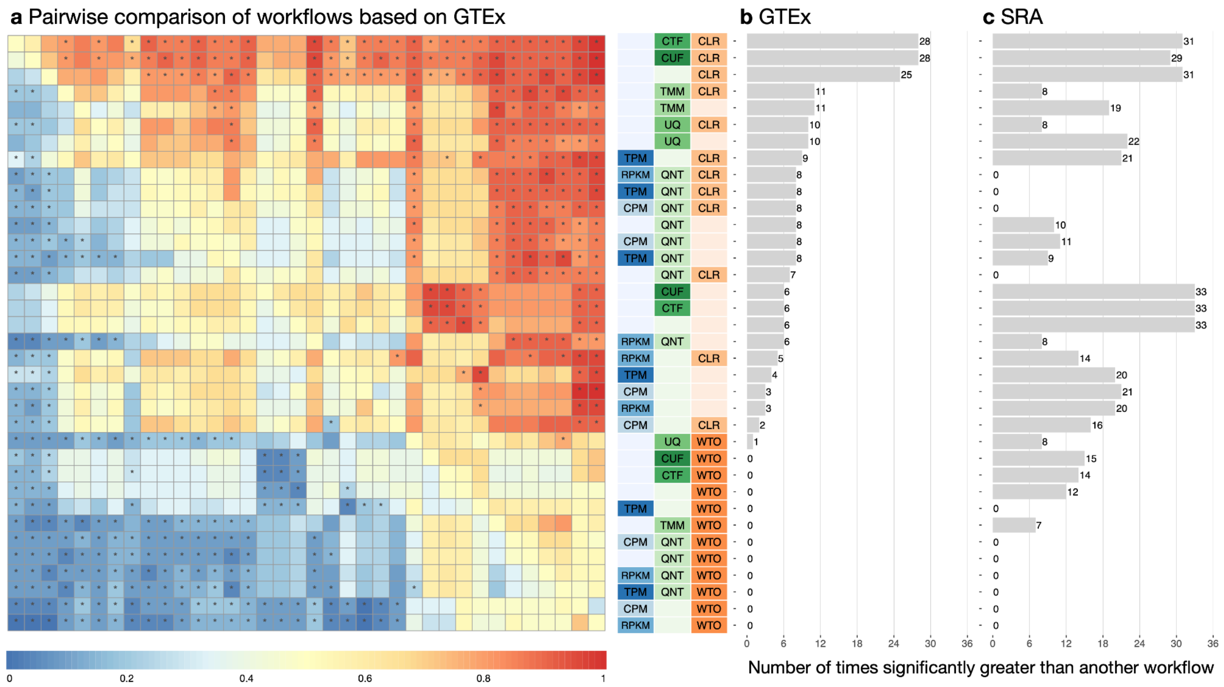

Figure S4. Dataset-level pairwise comparison of workflow performance for GTEx and SRA datasets based on the tissue-aware gold standard. (**a**) The heatmap shows the relative performance of a pair of workflows, corresponding to a row and a column, directly compared to each other for the GTEx datasets based on the tissue-aware gold standard. The color in each cell (row, column) represents the proportion of datasets for which the workflow along the row has a higher log2(auPRC/prior) than the workflow along the column. Comparisons that are statistically significant (corrected $p < 0.01$) based on a paired Wilcoxon test are marked with an asterisk. *Figures S4* contains the corresponding heatmap for the SRA datasets. (**b** and **c**) Barplots show the number of times each workflow was significantly greater than another workflow for GTEx (left) and SRA (right) datasets. Figure 3 and S3 contain these performance plots based on the tissue-naive gold standard.

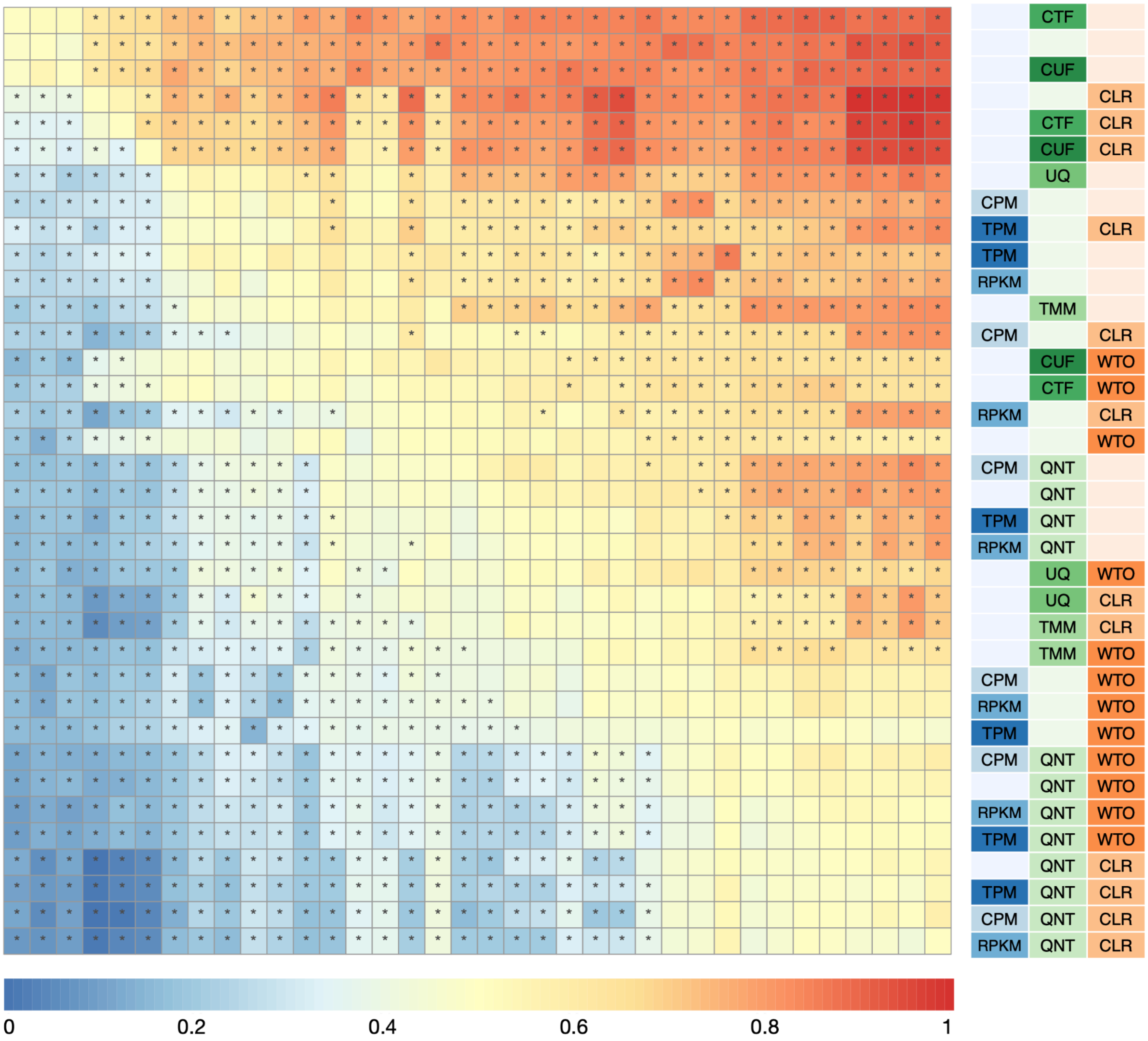

Figure S5. Dataset-level pairwise comparison of workflow performance for SRA datasets based on the tissue-aware gold standard. The heatmap shows the relative performance of a pair of workflows, corresponding to a row and a column, directly compared to each other for the SRA datasets based on the tissue-aware gold standard. The color in each cell (row, column) represents the proportion of datasets for which the workflow along the row has a higher log2(auPRC/prior) than the workflow along the column. Comparisons that are statistically significant (corrected $p < 0.01$) based on a paired Wilcoxon test are marked with an asterisk. *Figure S4a* contains the corresponding heatmap for GTEx datasets.

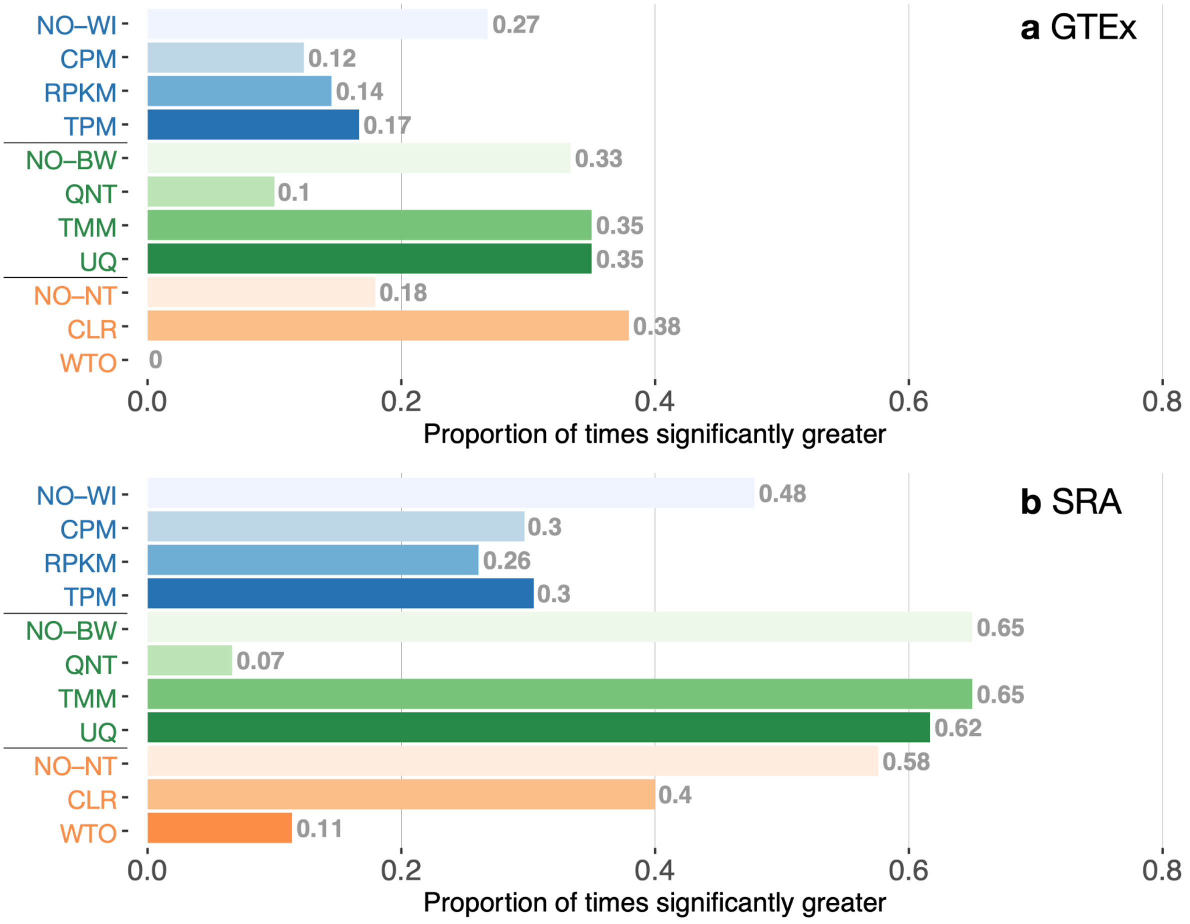

Figure S6. Impact of individual methods on performance of workflows based on the tissue-aware gold standard. Each bar in the two barplots, corresponding to a specific method, shows the proportion of times (x-axis) that workflows including that particular method (y-axis) were significantly better than other workflows. The barplots correspond to performance for the (**a**) GTEx and (**b**) SRA datasets evaluated on the tissue-naive gold standard. In order to make the comparison of between-sample normalization methods fair, workflows including CPM, RPKM, or TPM were left out because it is not possible to pair them with TMM or UQ normalization. Similarly, TMM and UQ methods are not included for "no within-sample normalization" (NO–WI). *Figure 4* contains these barplots based on the tissue-naive gold standard.

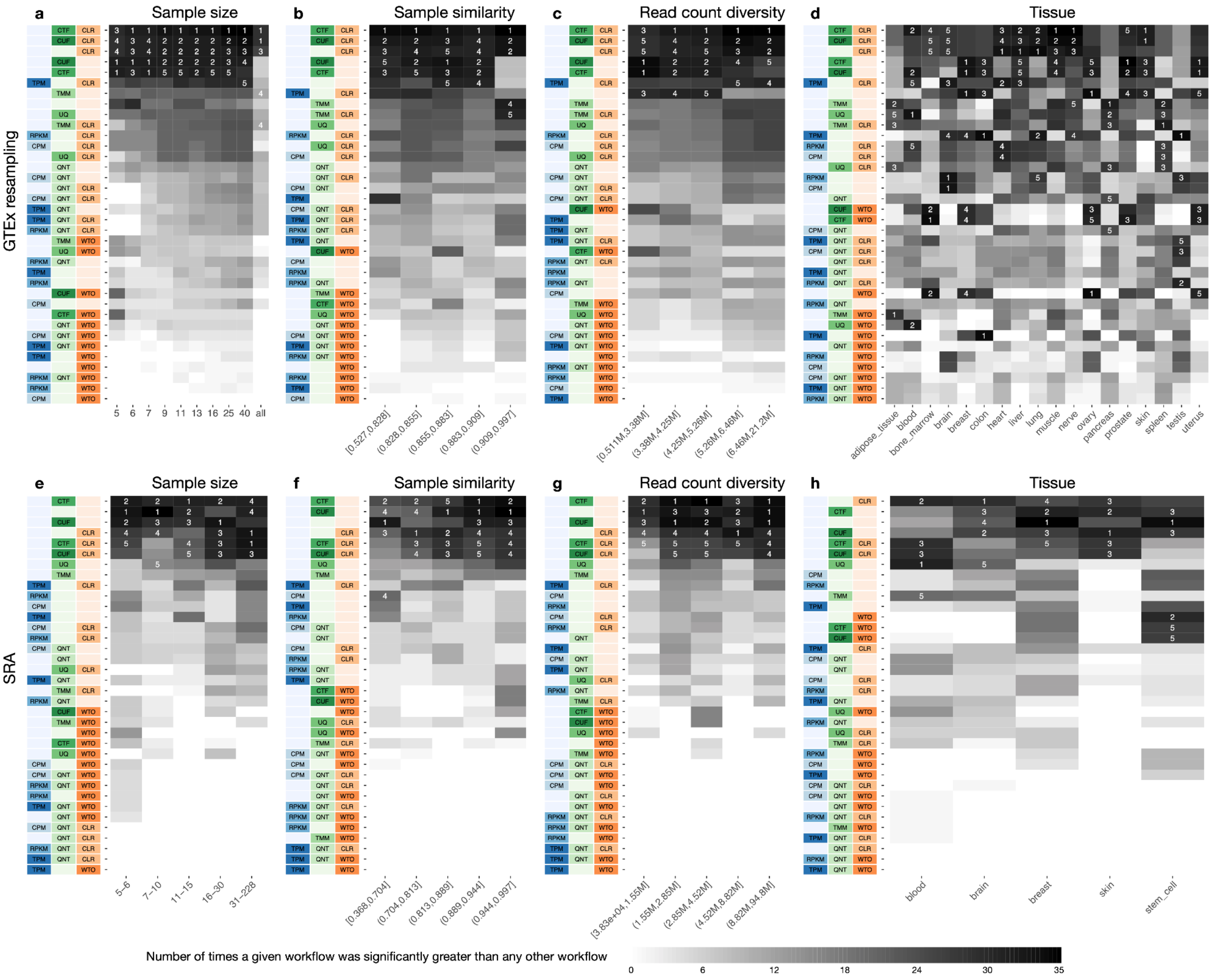
a
Sample size
b
Sample similarity
c
Read count diversity
d
Tissue
GTEx resampling
e
f
g
h
SRA
Number of times a given workflow was significantly greater than any other workflow

Figure S7. Impact of various dataset-related experimental factors on performance of workflows based on the tissue-aware gold standard. Each heatmap shows the number of times (cell color) each workflow (row) outperforms other workflows as a particular experimental factor pertaining to the input datasets is varied (columns), when the resulting coexpression networks are evaluated based on the tissue-naive gold standard. The darkest colors indicate workflows that are significantly better than the most other workflows. In addition, the top 5 workflows in each column are marked with their rank, with ties given minimum rank. The heatmaps on the top (**a**–**d**) correspond to datasets from GTEx resampling and those on the bottom (**e**–**h**) correspond to SRA datasets. The heatmaps from left to right show workflow performance by sample size (**a**, **e**; number of samples used to make the coexpression network), sample similarity (**b**, **f**; median spearman correlation of 50% most variable genes between samples), library size diversity by counts (**c**, **f**; standard deviation of counts sums across samples), and tissue of origin (**d**, **h**). *Figure 5* contains these heatmaps based on the tissue-naive gold standard.

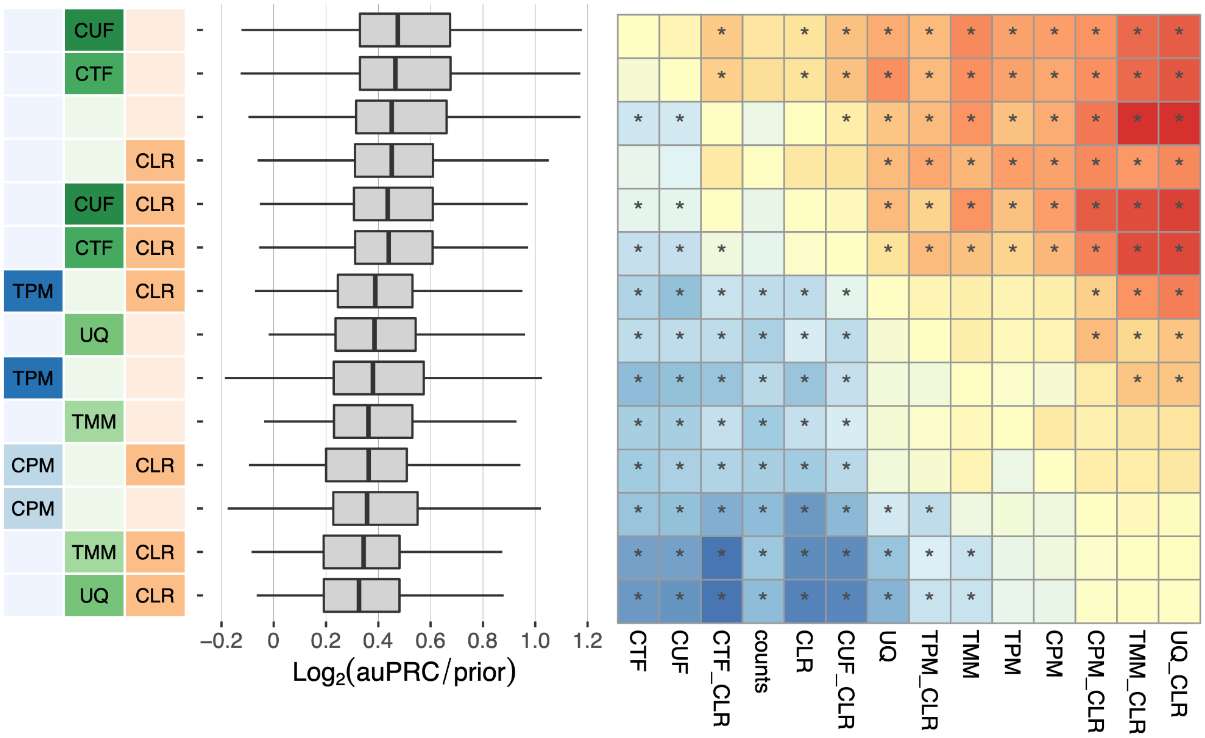

Figure S8. Overall performance of workflows and pairwise-comparison using refine.bio datasets based on the tissue-aware gold standard. The boxplots show the aggregate accuracy of all coexpression networks resulting from each individual workflow using SRA datasets in refine.bio, evaluated based on the tissue-aware gold standard. The performance of each workflow is presented as boxplots (without outliers) that summarizes the log2(auPRC/prior) of each workflow where auPRC is the area under the precision recall curve (see *Methods*). The workflows are ordered by their median log2(auPRC/prior). The heatmap shows the relative performance of pairs of workflows (rows and columns) directly compared to each other for the refine.bio SRA datasets based on the tissue-aware gold standard. The color in each cell (row, column) represents the proportion of datasets for which the workflow along the row has a higher log2(auPRC/prior) than the workflow along the column. Comparisons that are statistically significant (corrected $p < 0.01$) based on a paired Wilcoxon test are marked with an asterisk. *Figure 6* contains these plots based on the tissue-naive gold standard.

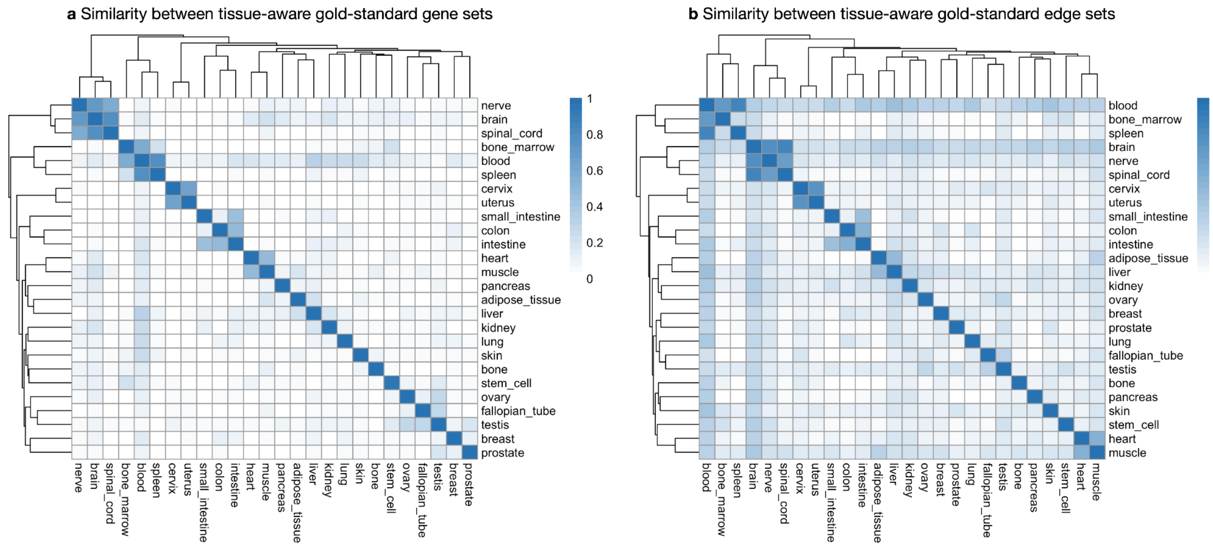

Figure S9. Gene- and edge-based overlap between tissue-aware gold standards. The heatmaps show the number of (**a**) genes or (**b**) edges that are shared between any two given tissue-aware gold standards divided by the total number of genes or edges in the smaller of the two tissue-aware gold standards. Based on the heatmaps, the proportion of shared genes and edges between unrelated tissues is small and therefore each tissue-aware gold standard is evaluating a very different set of biological relationships.

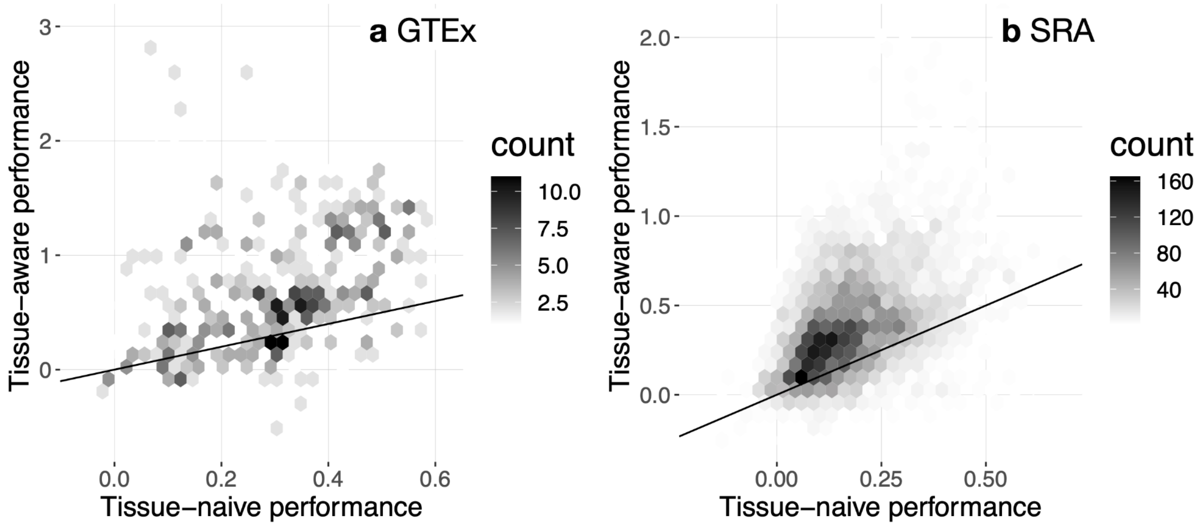

Figure S10. Overall accuracy of coexpression networks when evaluated based on the tissue-naive and tissue-aware gold standards. Each density plot – for the (**a**) GTEx and (**b**) SRA datasets – shows the distribution of log2(auPRC/prior) across all workflows and datasets when evaluating based on the tissue-naive gold standard (x-axis) vs. the tissue-aware gold standard (y-axis). These distributions show that coexpression networks capture tissue-aware gene interactions and emphasises the importance of evaluating coexpression networks using tissue-aware gold standards.

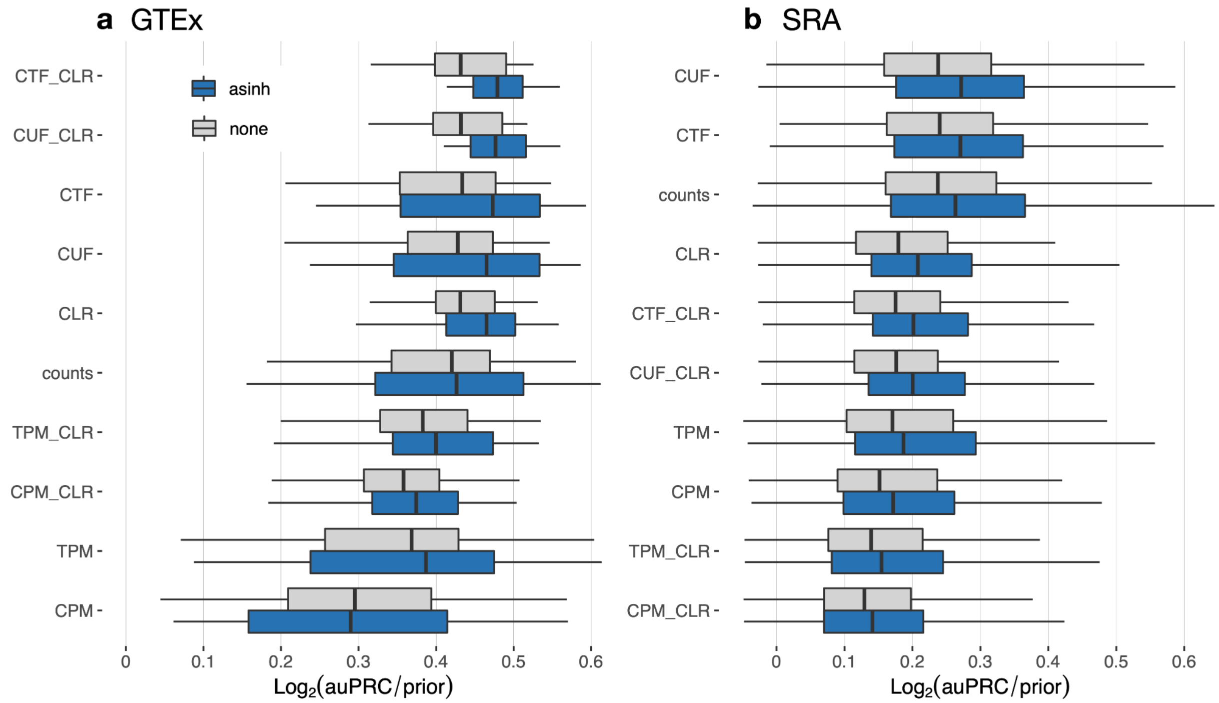

Figure S11. Overall performance of top workflows with and without asinh transformation based on the tissue-naive gold standard. The plots show the aggregate accuracy of all coexpression networks resulting from the top ten individual workflows using (**a**) GTEx and (**b**) SRA datasets with (blue) and without (gray) the asinh transformation, evaluated based on the tissue-naive gold standard. The workflows (rows) are described in terms of the specific method used in the within-sample normalization, between-sample normalization, and network transformation stages. The performance of each workflow is presented as boxplots (without outliers) that summarizes the log2(auPRC/prior) of each workflow where auPRC is the area under the precision recall curve (see Methods). The workflows are ordered by their median log2(auPRC/prior) in each panel.

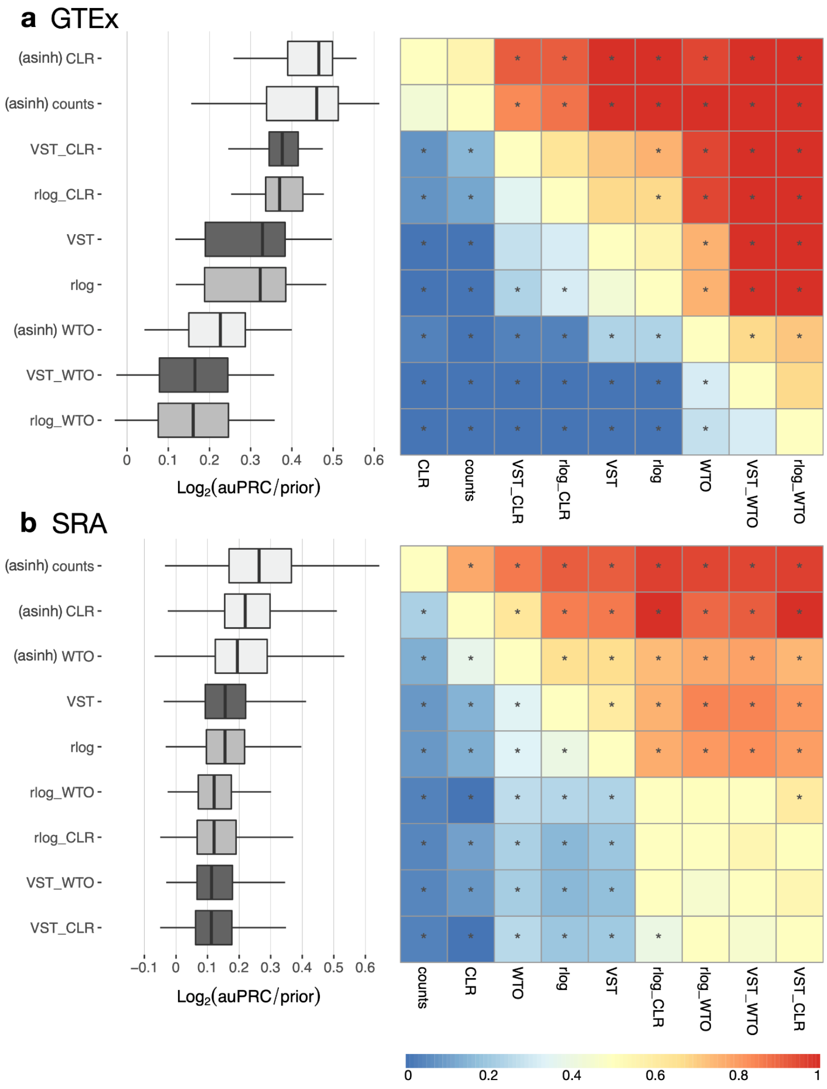

Figure S12. Performance of workflows using different data transformation methods based on the tissue-naive gold standard. The plots show the aggregate accuracy of all coexpression networks resulting from using (**a**) GTEx and (**b**) SRA datasets with different data transformations to adjust gene counts paired with the network transformation methods, evaluated based on the tissue-naive gold standard. The workflows (rows) are combinations of specific data transformations (shades of gray) and network transformations. The performance of each workflow is presented as boxplots (without outliers) that summarize the log2(auPRC/prior) of each workflow where auPRC is the area under the precision recall curve (see *Methods*). The workflows are ordered by their median log2(auPRC/prior) in each panel. The heatmaps on the right show the relative performance of a pair of workflows, corresponding to a row and a column, directly compared to each other for the GTEx (**a**) and SRA (**b**) datasets based on the tissue-naive gold standard. The color in each cell (row, column) represents the proportion of datasets for which the workflow along the row has a higher log2(auPRC/prior) than the workflow along the column. Comparisons that are statistically significant (corrected $p < 0.01$) based on a paired Wilcoxon test are marked with an asterisk. The six largest GTEx datasets (adipose_tissue, blood, blood_vessel, brain, esophagus, and skin) are not considered in this evaluation because of the considerable amount of computing time required to use rlog transformation on large datasets. *CLR* and *Counts* significantly outperformed all other methods on GTEx datasets. For SRA datasets, *Counts* performed significantly better than all other workflows, and *CLR* and *WTO* both performed significantly better than all workflows incorporating VST or rlog.

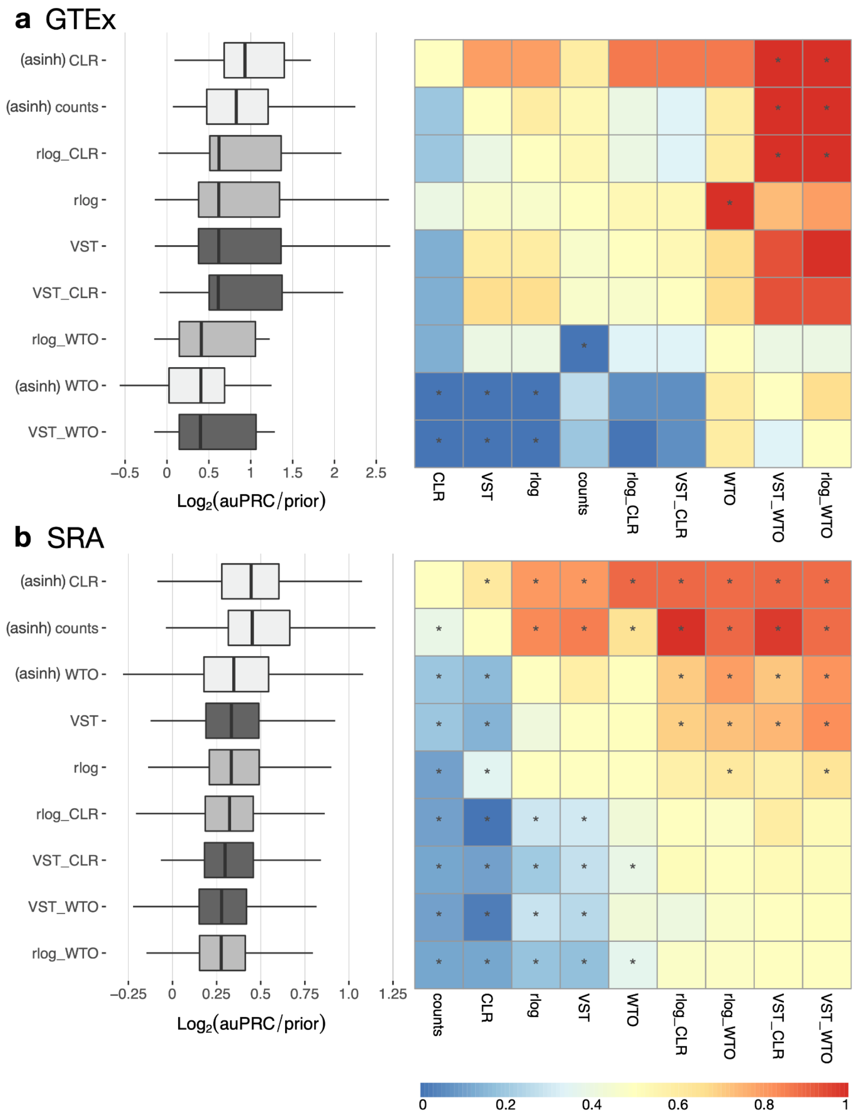

Figure S13. Performance of workflows using different data transformation methods based on the tissue-aware gold standard. The plots show the aggregate accuracy of all coexpression networks resulting from using (**a**) GTEx and (**b**) SRA datasets with different data transformations to adjust gene counts paired with the network transformation methods, evaluated based on the tissue-aware gold standard. The workflows (rows) are combinations of specific data transformations (shades of gray) and network transformations. The performance of each workflow is presented as boxplots (without outliers) that summarize the log2(auPRC/prior) of each workflow where auPRC is the area under the precision recall curve (see *Methods*). The workflows are ordered by their median log2(auPRC/prior) in each panel. The heatmaps on the right show the relative performance of a pair of workflows, corresponding to a row and a column, directly compared to each other for the GTEx (**a**) and SRA (**b**) datasets based on the tissue-aware gold standard. The color in each cell (row, column) represents the proportion of datasets for which the workflow along the row has a higher log2(auPRC/prior) than the workflow along the column. Comparisons that are statistically significant (corrected $p < 0.01$) based on a paired Wilcoxon test are marked with an asterisk. The largest GTEx datasets (adipose_tissue, blood, brain, and skin) are not considered in this evaluation because of the considerable amount of computing time required to use rlog transformation on large datasets. Fewer comparisons between workflows are statistically significant when evaluated on the tissue-aware gold standard, but *CLR* and *Counts* remain top performing methods for both GTEx and SRA datasets.

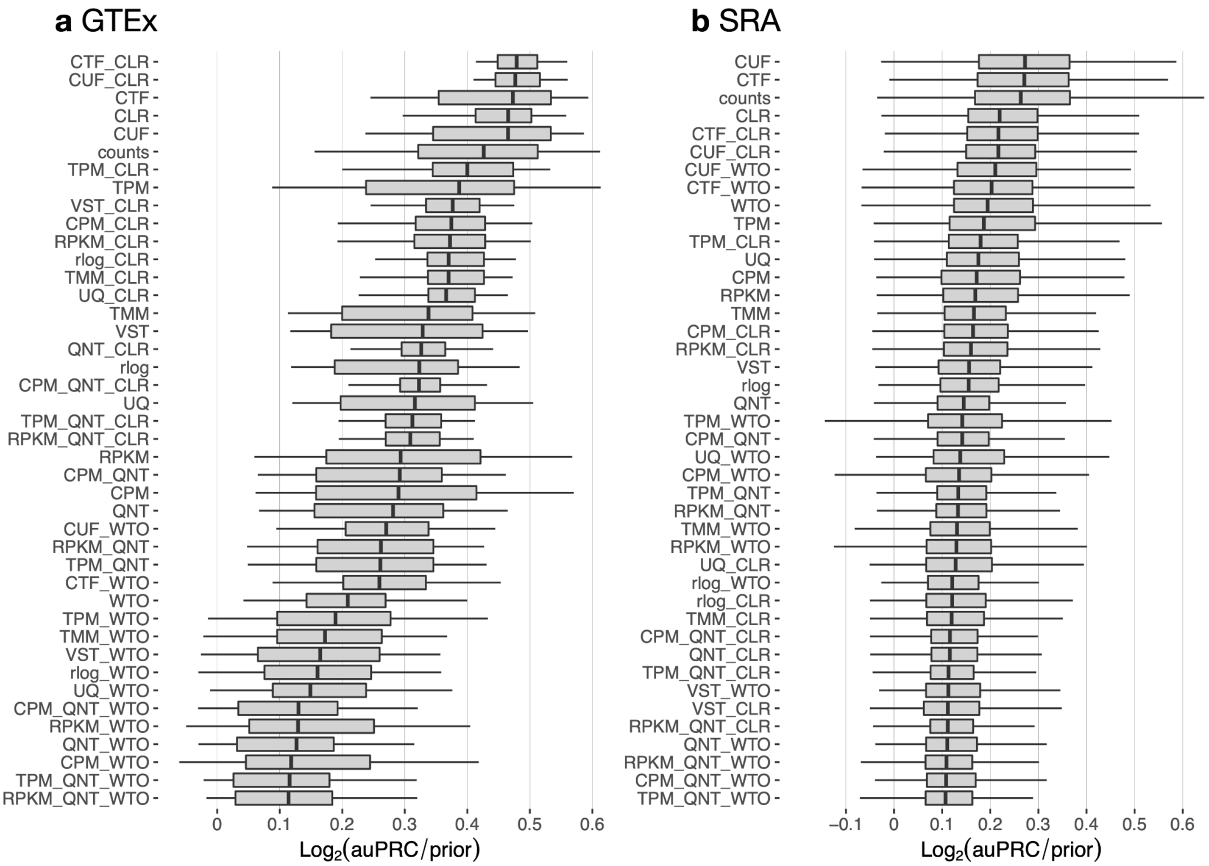

Figure S14. Overall performance of workflows based on the tissue-naive gold standard. The plots show the aggregate accuracy of all coexpression networks resulting from each individual workflow using (**a**) GTEx and (**b**) SRA datasets, evaluated based on the tissue-naive gold standard. The workflows (rows) are described in terms of the specific method used in the within-sample normalization, between-sample normalization, data transformation, and network transformation stages. The performance of each workflow is presented as boxplots (without outliers) that summarizes the log2(auPRC/prior) of each workflow where auPRC is the area under the precision recall curve (see Methods). The workflows are ordered by their median log2(auPRC/prior) for each panel. The six largest GTEx datasets (adipose_tissue, blood, blood_vessel, brain, esophagus, and skin) are not considered in this evaluation because of the considerable amount of computing time required to use rlog transformation on large datasets.

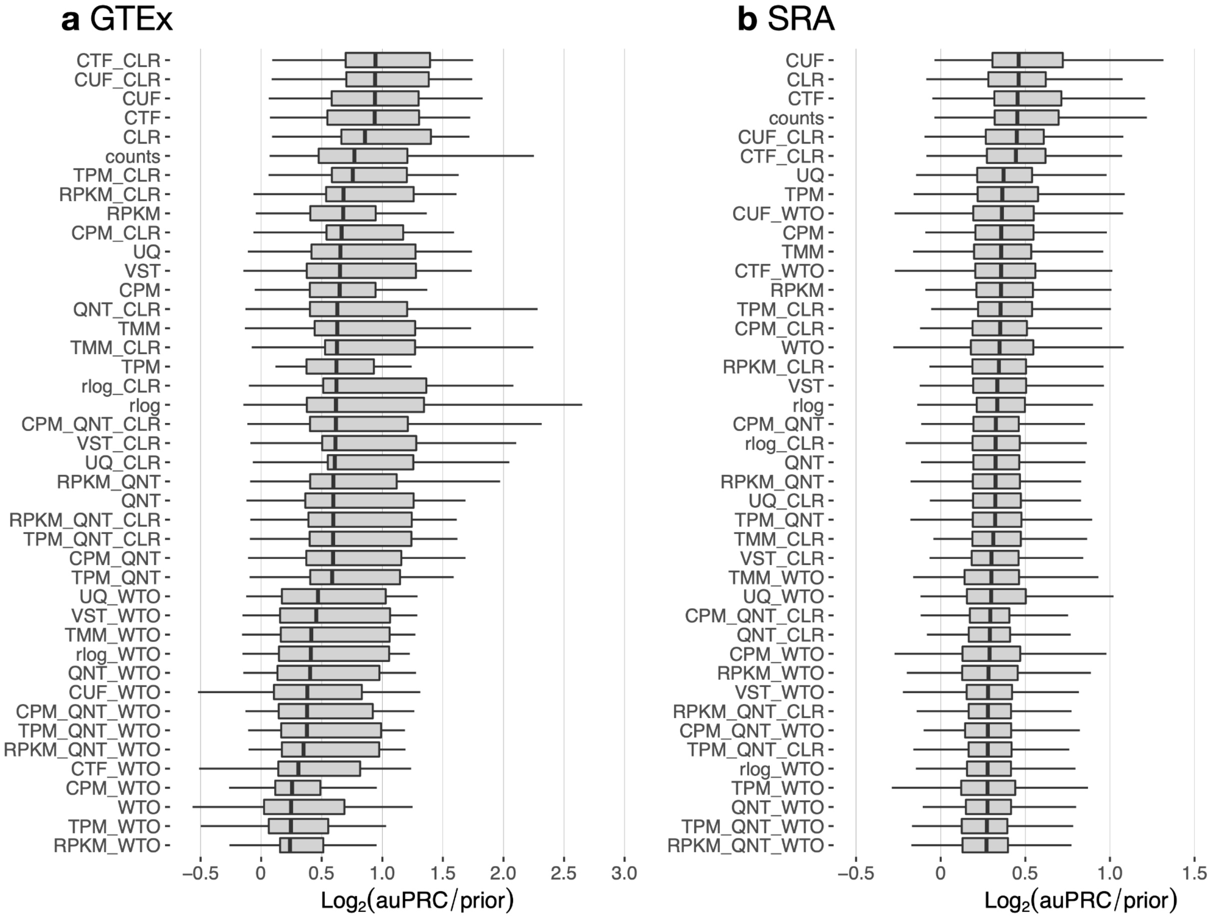

Figure S15. Overall performance of workflows based on the tissue-aware gold standard. The plots show the aggregate accuracy of all coexpression networks resulting from each individual workflow using (**a**) GTEx and (**b**) SRA datasets, evaluated based on the tissue-aware gold standard. The workflows (rows) are described in terms of the specific method used in the within-sample normalization, between-sample normalization, data transformation, and network transformation stages. The performance of each workflow is presented as boxplots (without outliers) that summarizes the log2(auPRC/prior) of each workflow where auPRC is the area under the precision recall curve (see Methods). The workflows are ordered by their median log2(auPRC/prior) in each panel.

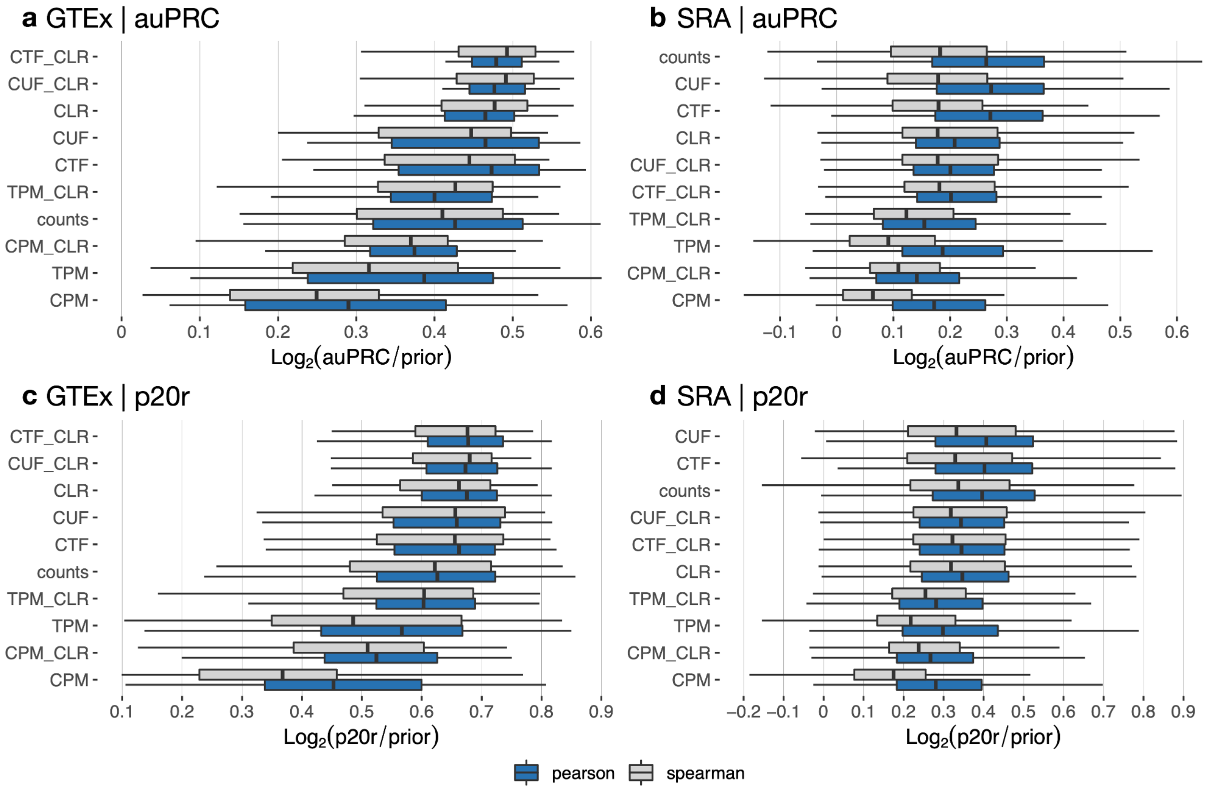

Figure S16. Overall performance of top ten workflows using Pearson and Spearman correlation based on the tissue-naive gold standard. The plots show the aggregate accuracy of all coexpression networks resulting from the top ten individual workflows using Pearson (blue) or Spearman (gray) correlation to build the network using (**a**, **c**) GTEx and (**b**, **d**) SRA datasets, evaluated based on the tissue-naive gold standard. The workflows (rows) are described in terms of the specific method used in the within-sample normalization, between-sample normalization, and network transformation stages. The performance of each workflow is presented as boxplots (without outliers) that summarizes the log2(auPRC/prior) (**a**, **b**) or the log2(p20r/prior) (**c**, **d**) of each workflow where auPRC is the area under the precision recall curve and p20r is the precision at 20% recall (see *Methods*). The workflows are ordered by their median log2(auPRC/prior) in each panel. Pearson correlation clearly yields better performance in all cases for the SRA data (i.e. datasets typically generated by individual research labs). Pearson also usually yields better results for the GTEx data as well, and more so when considering the accuracy of the top-scoring edges (evaluated using p20r).

Supplemental Note

Rationale for our functional gold standard
The definition of the “true” network structure is a crucial aspect when evaluating the accuracy of any network, including a coexpression network. Our choice and design of this ground-truth using GO biological process annotations is based on a number of factors including: A) many prior studies that link coexpression to GO co-annotation, B) the applications of coexpression networks for function prediction, and C) several previous studies that have established the strength and utility of GO-based ground-truth.

A) Prior studies link coexpression to GO co-annotation.
From the conception of high-throughput gene-expression techniques, studies have shown that coexpression between genes can be productively and accurately used to separate genes into functional modules [1, 2]. A number of other studies have explicitly tested the coexpression–co-annotation hypothesis and have shown that coexpressed genes are highly likely to be transcriptionally co-regulated and are often functionally related to each other by virtue of taking part in the same biological process or physiological trait [3, 4].

B) Coexpression is commonly used to study gene function.
Gene function prediction and gene module detection are the two major and most common applications of coexpression networks. These applications are based on the fact that functionally-related gene pairs or groups (i.e. members of a specific biological pathway or process) tend to be coexpressed with each other in high-throughput gene-expression datasets. By inverting this association, coexpression networks have often been successfully used in the literature to predict gene function and pathway membership [5]. Further, coexpression networks are frequently used to identify functional modules (i.e. entire pathways/processes) by clustering the network and performing GO-based functional enrichment on each cluster of genes [6]. Therefore, to assess the workflows examined in this study in relation to these most common applications, we chose to evaluate the accuracy of the resulting coexpression networks based on their ability to recapitulate gene functional relationships.

C) Strength of GO-based ground-truth of gene functional relationships.
Since functionally-related genes tend to be coexpressed with each other (A) and coexpression networks are routinely used to infer gene function and pathway/process membership (B), we reasoned that it would be most appropriate to evaluate the accuracy of coexpression networks based on their ability to recapitulate gene functional relationships based on their co-annotations to GO biological processes (GOBP).

However, creating a ground-truth about gene functional relationships (gold standard) from GO BP is not straightforward and should be done very carefully. For example, the Gene Ontology has many generic terms for biological processes such as “metabolism” or “stress response”. For

such terms, it would indeed be incorrect to assume that genes that are co-annotated to any of these terms should be connected in the co-expression network. Therefore, we have devised a careful procedure for constructing a gene functional gold standard based on GOBP. First, we do not use all GOBP terms to construct our gold standard. Following previous work [7], we use only 607 "specific" GOBP terms. These terms were selected by a team of seven graduate students and postdocs (with training in cell/molecular biology and genetics) based on the following procedure: To select "specific" terms, this question was considered: "if unknown gene/protein G were predicted to be annotated to GO term T, would that be enough to consider experimentally testing this relationship between G and T?". Only terms that were declared "yes" by the majority were retained as specific terms and only gene pairs co-annotated to any of these specific terms – based on experimental evidence – are considered to have a positive relationship in our gold standard. Similarly, assuming every other gene pair is a negative (not functionally related) would be far too strong an assumption. So, the team also selected a set of 75 "intermediate" GOBP terms such as "protein folding" or "cell proliferation". Then, to be considered as a negative in the gold standard, gene pairs must meet the following three criteria:

1. The two genes are *not* co-annotated to any intermediate term
2. The two genes are *not* co-annotated to significantly overlapping specific terms (hypergeometric test; p-value <0.05)
3. Each gene individually has at least one annotation to a specific term

Gene pairs that are co-annotated to intermediate terms (criterion 1) would be considered too close in general function to be sure that they are *not* functionally related. The hypergeometric test (criterion 2) prevents gene pairs that may share a function (due to being annotated to two overlapping terms) from being labeled as negatives. Requiring a specific term annotation for each gene ensures that no assumptions are made about genes that have not been experimentally studied before.

This procedure for creating GOBP-based gold standards of gene functional relationships has several advantages, which are outlined in detail in the paper that first used this procedure to create a functional gold standard for yeast [8]. Briefly, the advantages of this gold standard over other options include lack of substantial functional bias, lack of varying specificity problems, a thoughtful method of defining negatives, and a more proportional ratio of positive and negative examples. Careful manual selection of specific terms covers the first two issues. Using all terms in GOBP or all pathways in a different database results in functional biases towards very large pathways which can 'make or break' the evaluation (see the first figure and ribosome KEGG pathway in [9]). Alternatively, defining a 'specificity cutoff' for these ontology structures (whether by number of annotations or depth in the ontology) results in wildly different biological specificity of terms (Figure 3, in [8]). Finally, manual selection of intermediate terms in our gold standard procedure allows negatives to be defined sensically and confidently with enough pairs to far outnumber the positive examples. This reflects the ground truth, which is that there are far more pairs of genes that do not interact with each other than gene pairs that do interact with each other.

In summary, the definition of the ground-truth network structure (i.e., the gold standard) is based on: previously established observations about the connection between coexpression and functional co-annotation; the applications of coexpression to delineate gene function; and our rigorous procedure for setting up a meaningful set of functionally-related and unrelated gene pairs based on experimental annotations of genes to specific terms in GOBP.

Other gold standards considered

We spent a considerable amount of effort trying to create other gold standards without much success due to the lack of appropriate external datasets. For example, we attempted to create gold standards based on groups of genes co-bound by the same transcription factor (in ChIP-Seq experiments). However, physical binding of transcription factor does not necessarily indicate functional interaction between the transcription factor and the target gene nor does it indicate co-regulation (coexpression) between the target genes. This limitation was apparent from our observation that coexpression networks evaluated on these TF-binding-based gold standards had random performance at best and worse than random performance otherwise, regardless of workflow, sample size, or data quality.

We also attempted to create a gold standard based on groups of genes co-annotated to only tissue-specific GO biological processes. However, there was very little experimental annotation in this data to create gold standards that span tens of thousands of genes for many tissues.

At least one previous study has used spike-in data to construct ground-truth coexpression networks [9]. However, we did not choose to use spike-in data for reasons similar to the ones outlined above: limited data that prevents conducting an evaluation on a large-enough scale to be comfortable drawing general conclusions. To our knowledge, there is not a large collection of readily-available spike-in data from multiple sources and tissues to leverage for this purpose. RNA-seq experiments are quite sensitive to technical effects when considering final quantification of each gene count in a given sample. These technical effects are a combination of the specific transcripts of interest (GC content, length, reverse transcriptase binding site sequence, etc) and the overall distribution of the population of RNA/cDNA in the sample library (whether rRNA depletion or polyA+ tail selection is used, which tissue the sample comes from, sequencing protocol, etc). All these factors can have significant effects on total read counts and, thus, gene count quantification. And, spike-in controls are not immune to these effects [10]. As we discuss throughout the paper, different normalization and network transformation techniques handle these technical biases differently, whether explicitly or implicitly. Therefore, to call a method or workflow “robust”, it must work well over a large number of datasets that encompass datasets with any number of a variety of technical biases. In our study, we check for robustness by using a large number of primary bulk RNA-seq samples (over 15,000) from over 35 tissues and 200 independent studies that were all quantified into gene counts by the same alignment software. It would be a considerable effort to collect data with spike-ins from many sources and process the raw reads into counts data for another large set of data, as we used Recount2 to take away the variability of using different alignment software. Currently, there does not seem to be a single, high-quality, large dataset for spike-in RNA-seq data that would correspond to

something like the data used in our GTEx analysis where the raw reads are converted to gene counts using the same quality-control and quantification procedure (like in Recount2) to take away the variability of using different alignment software.

Furthermore, even if there is enough spike-in data available, it is not certain that it would be a useful evaluation. The purpose of spike-in experiments is often to estimate the precision and accuracy of the sequencing technology. So, typically, the concentration of the spike-in is *equal* across samples in a dataset. Even in cases where the concentration of a given spike-in probe is varied across samples, the point of these spike-ins is to find the limit of detection or to be at a detectable level so that they can be used for quantification. This means that, in many cases, we would only be able to evaluate spike-in oligos with a nominal correlation of one or zero (as was done in [9]). To be clear, this means that we would not be able to evaluate any correlation between 0 and 1. This limitation skews the assessment of workflows to an evaluation of genes that are perfectly coexpressed and highly 'expressed' in at least some samples. As discussed briefly in the *Discussion* section, the mean-correlation relationship bias (the observation that highly-expressed genes tend to be more highly-coexpressed in coexpression analysis) might make this type of gold standard rather easy to achieve for all workflows. Such a gold standard does not represent a large number of genes that are never highly expressed but are nonetheless genes of great interest.

Evaluation procedure using our gold standard

The gold standard contains thousands of gene pairs that either have a functional relationship (positive) or do not have a functional relationship (negative), defined based on experimental gene co-annotations to specific GOBP terms (see above). Then, we evaluate each coexpression network (derived from a single RNA-seq dataset analyzed using any one of the workflows) by comparing it to this gold standard by essentially asking the following question: do gene pairs that have very high coexpression strengths (i.e. high correlation coefficients) tend be functionally related to each other based on the gold standard?

We answer this question quantitatively by calculating the area under the precision-recall curve for that coexpression network in the following manner:

1. We rank all the gene pairs in the network from highest to lowest correlation.
2. Then, at various cutoffs of correlation strength from high to low, we calculate the number of true positives, false positives, true negatives, and false negatives.
    a. Gene pairs with a correlation value above the cutoff and,
        i. Functionally related in the gold standard (i.e., positive) are 'true positives' (TP).
        ii. Not functionally related in the gold standard (i.e., negative) are 'false positives' (FP).
    b. Gene pairs with a correlation value below the cutoff and,
        i. Functionally related in the gold standard (i.e., positive) are 'false negatives' (FN).
        ii. Not functionally related in the gold standard (i.e., negative) are 'true negatives' (TN).
    c. These TP, FP, FN, and TN values are combined to calculate the precision (= TP / (TP + FP)) and recall (= TP / (TP + FN)) at that cutoff.
3. All the precision and recall values at the various correlation cutoffs are used together to build the precision-recall curve.

4. Finally, the area under this curve (auPRC) and the precision that corresponds to 20% recall (p20r) are used to quantify the ability of the coexpression network to recapitulate gene functional relationships in the gold standard.

References

1. Eisen MB, Spellman PT, Brown PO, and Botstein D. Cluster analysis and display of genome-wide expression patterns. PNAS 1998. Link
2. Segal E, Friedman N, Koller D, Regev. A module map showing conditional activity of expression modules in cancer. Nature Genetics 2004. Link
3. Allocco DJ, Kohane IS, and Butte AJ. Quantifying the relationship between co-expression, co-regulation and gene function. BMC Bioinformatics 2004. Link
4. Carpenter AE and Sabatini DM. Systematic genome-wide screens of gene function. Nature reviews genetics 2004. Link
5. Zhu Q, Wong AK, Krishnan A, Aure MR, Tadych A, Zhang R, Corney DC, Greene CS, Bongo LA, Kristensen VN, Charikar M, Li K and Troyanskaya OG. Targeted exploration and analysis of large cross-platform human transcriptomic compendia. Nature Methods 2015. Link
6. Zhang B, Horvath S. A General Framework for Weighted Gene Co-Expression Network Analysis. Stat Appl Genet Mol Biol. De Gruyter; 2005;4. Link
7. Greene CS, Krishnan A, Wong AK, Ricciotti E, Zelaya RA, Himmelstein DS, Zhang R, Hartmann BM, Zaslavsky E, Sealfon SC, Chasman DI, FitzGerald GA, Dolinski K, Grosser T and Troyanskaya OG. Understanding multicellular function and disease with human tissue-specific networks. Nature Genetics 2015. Link
8. Myers CL, Barrett DR, Hibbs MA, Huttenhower C and Troyanskaya OG. Finding function: evaluation methods for functional genomic data. BMC Genomics 2006. Link
9. McCall MN and Almudevar A. Affymetrix GeneChip microarray preprocessing for multivariate analyses. Briefings in Bioinformatics 2012. Link
10. Qing T, Yu Y, Du T T, et al. mRNA enrichment protocols determine the quantification characteristics of external RNA spike-in controls in RNA-Seq studies. Sci China Life Sci, 2013, 56: 134–142. Link
