# Supplementary material for: Robust normalization and transformation techniques for constructing gene coexpression networks from RNA-seq data
Source: Genome Biol. 2022 Jan 3;23:1. doi: 10.1186/s13059-021-02568-9 (PMC8721966; doi:10.1186/s13059-021-02568-9)
Supplement: Supplementary file 2 — Additional file 2. Review history. [file 13059_2021_2568_MOESM2_ESM.docx]

Review History

**First round of review**

**Reviewer 1**

**Are you able to assess all statistics in the manuscript, including the appropriateness of statistical tests used?**

Yes, and I have assessed the statistics in my report.

**Comments to author:**

The authors describe a benchmark study examining RNA-seq preprocessing and analysis workflows focusing on co-expression network estimation. This is certainly an important and timely area of inquiry, given the vast amount of RNA-seq data being made available and the ongoing evolution of our understanding of the diverse sources of covariation captured by co-expression networks. However, in my opinion, this study is limited in scope and relies too heavily on GO annotation to assess the preprocessing workflows. Specific comments are below.

Major Compulsory Revisions

1. The definition of the true network structure used in the calculation of precision and recall, what the authors refer to as the gold standard, represents a substantial limitation to the interpretation of the results. Specifically, the authors assume that genes that share a GO biological process term should be connected in the co-expression network. This is a strong assumption that largely goes unexamined / untested. Furthermore, the tissue-specific networks were simply created by subsetting the genes expressed in that tissue. This assumes that there is no tissue-specific regulation.

2. While the authors exhaustively compare the different workflows based on the area under the precision recall curve using the GO annotation based "true" networks, I expected to see additional supporting analyses using other approaches to get at a "true" or expected network. For example, one could use spike-ins or mixture / dilution data, as was previous done for microarray data in:

McCall, Matthew N., and Anthony Almudevar. "Affymetrix GeneChip microarray preprocessing for multivariate analyses." Briefings in bioinformatics 13.5 (2012): 536-546.

Minor Essential Revisions

1. While the results of the study could also be found on the website:

https://krishnanlab.github.io/RNAseq_coexpression

I was not able to find code to reproduce the analyses.

**Reviewer 2**

**Are you able to assess all statistics in the manuscript, including the appropriateness of statistical tests used?**

Yes, and I have assessed the statistics in my report.

**Comments to author:**

The paper aims at tackling the relevant and interesting challenge of how to process RNA-seq data with the goal of constructing co-expression networks. This is as of yet unclear, and the paper therefore aims to fill an important gap in the literature. I therefore think the topic itself is interesting, but I feel that the authors have missed several opportunities in this paper that would significantly improve it.

Major comments

-	In the Background section, the authors discuss the terms ‘normalization’ and ‘transformation’. At first read, I had considered normalization to stand for within-/between-sample normalization such as CPM or TMM, and transformation to stand for transformation of the data, such as log-transformation. It turns out the authors restrict the use of the word ‘transformation’ to transform the obtained co-expression network. The authors should more clearly differentiate between these two terms. A log-transformation of the data can be considered a normalization; these two terms are not necessarily separate ideas (as they seem to be considered in the manuscript) of working with the data and the Background section would benefit by, from the start on, clearly mentioning what the authors mean when they use these words.

-	The authors evaluate a range of pre-processing methods to evaluate the estimation of co-expression networks on a number of datasets. However, the authors do not release these obtained results as a resource to the community, which seems to be a missed opportunity. A consensus co-expression network as well as tissue-specific co-expression networks could be constructed and provided as valuable resources.

-	The gene filtering step removes genes which have less than one CPM in at least 80% of the samples in at least one dataset. Upon first reading, this looked stringent. Indeed, genes with tissue-specific expression would be removed when using this criterion (as they would not be expressed in a dataset not considering that tissue), significantly reducing the relevance of the tissue-specific co-expression evaluation. However, the authors still retain over 20K genes. Can the authors provide some intuition about this somewhat surprising result?

-	Several pre-processing steps must be made more clear / better argumented. At least, the robustness of the results should be assessed by including a ‘none’ option in each step, in particular the steps sample selection, gene filtering, gene type filtering, and hyperbolic arcisne. Some examples on unclarities:

o	The authors discuss ‘qauntile normalization’ (QNT). It is unclear whether this refers to ‘full-quantile normalization’ first adopted on RNA-seq data, I believe, by Bolstad et al. (2003), or some other form of quantile normalization.

o	TMM and UQ normalization produces offsets, which are often incorporated into GLMs for differential expression analysis. How are these offsets used here? Are normalized counts calculated using these offsets? Providing the equations for this seems necessary.

o	The preprocessing steps are introduced as being popular, but I personally have rarely read papers that use the hyperbolic arcsine transformation, and this does not seem to be a standard choice to me. The authors also do not argument it.

-	Tissue-specific gold standard networks used for evaluation are constructed by subsetting

the global gold standard network only using genes known to be expressed in a particular tissue. Is ‘known to be expressed in a particular tissue’ derived from the data, or a database? Also, this creation of tissue-specific gold standard networks does not seem to account for the fact that genes might be expressed in 2+ tissues, but differentially co-expressed in these different environments, which seems very relevant to evaluate tissue-specific co-expression. Can the authors comment on, and possibly rectify, this?

-	The authors evaluate some between-sample normalization methods, but the number of methods is still limited to three, and a ‘no normalization’ option. It might be useful to consider more options that are commonly used, e.g., DESeq2 normalization.

-	Networks are always constructed based on pairwise Pearson correlation between genes. There is an obvious missed opportunity to include co-expression measures in the evaluation benchmark. It would be a great contribution to study the interaction between pre-processing and evaluation metrics. Indeed, other work such as Teng et al. (2012) have demonstrated the clear disadvantages of using Pearson correlation on heavy-tailed count data such as RNA-seq datasets.

-	The authors did not provide code to reproduce the results in the manuscript. Please deposit your code in a relevant repository such that they may be browsed and re-used by other scientists.

-	Genes in the same GOBP term are positively co-expressed. However, asides from two exceptions relating to sets of genes that are co-expressed with many of the same genes or co-annotated in general GOBP terms, I interpret the writing that all other genes are decided upon to be negatively co-expressed. This seems like quite the stretch, as the authors seem to consider two independent genes to be negatively co-expressed, which can have drastic consequences to the evaluation procedure.

-	It is unclear to me what the authors consider as a ‘true positive’, ‘false positive’ etc. Do the authors consider a true positive as soon as the sign of the Pearson correlation coefficient matches the sign of the edge in the gold standard? Is significance considered? Multiple testing?

General comments

-	No preprocessing at all, neither on the data nor the co-expression network, performs best in the SRA evaluation. This is pretty surprising, and should be discussed and explored. It is also the sixth best method in the GTEx evaluation, and only performing CLR network transformation is the fourth best method, which is similarly pretty surprising. Even in datasets with a high read count diversity (Figure 5c,g), no between-sample normalization performs very well for GTEx and best for the SRA evaluation.

Minor comments

-	Table 1 would benefit from a ‘Number of experiments/datasets’ row.

-	The authors discuss the preprocessing procedure ‘counts’ which I assume is no within-sample normalization, no between-sample normalization and no network transformation, but this isn’t defined in the manuscript.

-	In the Methods: Network evaluation section, the authors write that they “assembled these gold standards by beginning with a set of manually-selected GOBP terms”. Some intuition on the previous work in [39] as to how/why these were selected would be useful.

-	The authors write that they got the tissue-specific genes from the TISSUES 2.0 database, and mention a z-score and Knowledge channel. It is unclear what is meant with this.

-	It is not mentioned how ‘sample similarity’ and ‘read count diversity’ are calculated and therefore the labels on the x-axis of Fig5 and related figures cannot be interpreted.

-	Genes that are annotated in the same GOBP term are considered to be positively co-

expressed, which seems reasonable. How are genes that are present in multiple GOBP terms handled?

**Authors Response**

**Point-by-point responses to the reviewers’ comments:**

Reviewer 1

=======

The authors describe a benchmark study examining RNA-seq preprocessing and analysis workflows focusing on co-expression network estimation. This is certainly an important and timely area of inquiry, given the vast amount of RNA-seq data being made available and the ongoing evolution of our understanding of the diverse sources of covariation captured by co-expression networks. However, in my opinion, this study is limited in scope and relies too heavily on GO annotation to assess the preprocessing workflows. Specific comments are below.

Major Compulsory Revisions

Comment 1.1

The definition of the true network structure used in the calculation of precision and recall, what the authors refer to as the gold standard, represents a substantial limitation to the interpretation of the results. Specifically, the authors assume that genes that share a GO biological process term should be connected in the co-expression network. This is a strong assumption that largely goes unexamined / untested.

*As the reviewer suspects, the definition of the “true” network structure is indeed a crucial aspect of any study on network reconstruction and evaluation and we have invested considerable thought and effort on this aspect. Below is a detailed account of the basis of the gold standard based on GO biological processes. We have now added this to a new Supplemental Note.*

*Our choice and design of this ground-truth using GO biological process annotations is based on a number of factors including: A) many prior studies that link coexpression to GO co-annotation, B) the applications of coexpression networks for function prediction, and C) several previous studies that have established the strength and utility of GO-based ground-truth.*

*A) Prior studies link coexpression to GO co-annotation.*

*From the conception of high-throughput gene-expression techniques, studies have shown that coexpression between genes can be productively and accurately used to separate genes into functional modules [Eisen et al.; Segal et al.]. A number of other studies have explicitly tested the coexpression–co-annotation hypothesis and have shown that coexpressed genes are highly likely to be transcriptionally co-regulated and are often functionally related to each other by virtue of taking part in the same biological process or physiological trait [Allocco et al., Carpenter and Sabatini].*

*B) Coexpression is commonly used to study gene function*

*Gene function prediction and gene module detection are the two major and most common applications of coexpression networks. These applications are based on the fact that functionally-related gene pairs or groups (i.e. members of a specific biological pathway or process) tend to be coexpressed with each other in high-throughput gene-expression datasets. By inverting this association, coexpression networks have often been successfully used in the literature to predict gene function and pathway membership [Zhu et al., Plaisier et al.]. Further, coexpression networks are frequently used to identify functional modules (i.e. entire pathways/processes) by clustering the network and performing GO-based functional enrichment on each cluster of genes [Langfelder and Horvath]. Therefore, to assess the workflows examined in this study in relation to these most common applications, we chose to evaluate the accuracy of the resulting coexpression networks based on their ability to recapitulate gene functional relationships.*

*C) Strength of GO-based ground-truth of gene functional relationships*

*Since functionally-related genes tend to be coexpressed with each other (A) and coexpression networks are routinely used to infer gene function and pathway/process membership (B), we reasoned that it would be most appropriate to evaluate the accuracy of coexpression networks based on their ability to recapitulate gene functional relationships based on their co-annotations to GO biological processes (GOBP).*

*However, creating a ground-truth about gene functional relationships (gold standard) from GO BP is not straightforward and should be done very carefully. For example, the Gene Ontology has many generic terms for biological processes such as “metabolism” or “stress response”. For such terms, it would indeed be incorrect to assume that genes that are co-annotated to any of these terms should be connected in the co-expression network.Therefore, we have devised a careful procedure for constructing a gene functional gold standard based on GOBP. First, we do not use all GOBP terms to construct our gold standard. Following previous work [Greene et al.], we use only 607 “specific” GOBP terms. These terms were selected by a team of seven graduate students and postdocs (with training in cell/molecular biology and genetics) based on the following procedure: To select “specific” terms, this question was considered: “if unknown gene/protein G were predicted to be annotated to GO term T, would that be enough to consider experimentally testing this relationship between G and T?”. Only terms that were declared “yes” by the majority were retained as specific terms and only gene pairs co-annotated to any of these specific terms – based on experimental evidence – are considered to have a positive relationship in our gold standard. Similarly, assuming every other gene pair is a negative (not functionally related) would be far too strong an assumption. So, the team also selected a set of 75 “intermediate” GOBP terms such as “protein folding” or “cell proliferation”. Then, to be considered as a negative in the gold standard, gene pairs must meet the following three criteria:*

*1. The two genes are not co-annotated to any intermediate term*

*2. The two genes are not co-annotated to significantly overlapping specific terms (hypergeometric test; p-value <0.05)*

*3. Each gene individually has at least one annotation to a specific term*

*Gene pairs that are co-annotated to intermediate terms (criterion 1) would be considered too close in general function to be sure that they are not functionally related. The hypergeometric test (criterion 2) prevents gene pairs that may share a function (due to being annotated to two overlapping terms) from being labeled as negatives. Requiring a specific term annotation for each gene ensures that no assumptions are made about genes that have not been experimentally studied before.*

*This procedure for creating GOBP-based gold standards of gene functional relationships has several advantages, which are outlined in detail in the paper that first used this procedure to create a functional gold standard for yeast [Myers et al.]. Briefly, the advantages of this gold standard over other options include lack of substantial functional bias, lack of varying specificity problems, a thoughtful method of defining negatives, and a more proportional ratio of positive and negative examples. Careful manual selection of specific terms covers the first two issues. Using all terms in GOBP or all pathways in a different database results in functional biases towards very large pathways which can ‘make or break’ the evaluation (see the first figure and ribosome KEGG pathway in Myers et al.). Alternatively, defining a ‘specificity cutoff’ for these ontology structures (whether by number of annotations or depth in the ontology) results in wildly different biological specificity of terms (Figure 3, supplemental in Myers et al.). Finally, manual selection of intermediate terms in our gold standard procedure allows negatives to be defined sensically and confidently with enough pairs to far outnumber the positive examples. This reflects the ground truth, which is that there are far more pairs of genes that do not interact with each other than gene pairs that do interact with each other.*

*In summary, the definition of the ground-truth network structure (i.e., the gold standard) is based on: previously established observations about the connection between coexpression and functional co-annotation; the applications of coexpression to delineate gene function; and our rigorous procedure for setting up a meaningful set of functionally-related and unrelated gene pairs based on experimental annotations of genes to specific terms in GOBP.*

*Eisen MB, Spellman PT, Brown PO, and Botstein D. Cluster analysis and display of genome-wide expression patterns. PNAS 1998. Link*

*Segal E, Friedman N, Koller D, Regev. A module map showing conditional activity of expression modules in cancer. Nature Genetics 2004. Link*

*Allocco DJ, Kohane IS, and Butte AJ. Quantifying the relationship between co-expression, co-regulation and gene function. BMC Bioinformatics 2004. Link*

*Carpenter AE and Sabatini DM. Systematic genome-wide screens of gene function. Nature reviews genetics 2004. link*

*Zhu Q, Wong AK, Krishnan A, Aure MR, Tadych A, Zhang R, Corney DC, Greene CS, Bongo LA, Kristensen VN, Charikar M, Li K and Troyanskaya OG. Targeted exploration and analysis of large cross-platform human transcriptomic compendia. Nature Methods 2015. Link*

*Plaisier CL, Horvath S, Huertas-Vazquez A, Cruz-Bautista I, Herrera MF, Tusie-Luna T, Aguilar-Salinas C, and Pajukanta P. A Systems Genetics Approach Implicates USF1, FADS3, and Other Causal Candidate Genes for Familial Combined Hyperlipidemia. PLOS Genetics 2009. Link*

*Langfelder P and Horvath S. WGCNA: an R package for weighted correlation network analysis. BMC Bioinformatics 2008. Link*

*Greene CS, Krishnan A, Wong AK, Ricciotti E, Zelaya RA, Himmelstein DS, Zhang R, Hartmann BM, Zaslavsky E, Sealfon SC, Chasman DI, FitzGerald GA, Dolinski K, Grosser T and Troyanskaya OG. Understanding multicellular function and disease with human tissue-specific networks. Nature Genetics 2015. Link*

*Myers CL, Barrett DR, Hibbs MA, Huttenhower C and Troyanskaya OG. Finding function: evaluation methods for functional genomic data. BMC Genomics 2006. Link*

Comment 1.2

Furthermore, the tissue-specific networks were simply created by subsetting the genes expressed in that tissue. This assumes that there is no tissue-specific regulation.

*We thank the reviewer for bringing up this fine point. First, to be more accurate, we have changed our “tissue-specific” evaluation to “tissue-aware” because we are only interested in whether a gene pair is functionally related in particular tissue or not (tissue-aware), and not whether that gene pair is only related in that tissue and not others (tissue-specific).*

*Second, the purpose of this evaluation is to make sure that as many networks as possible were also being evaluated on their ability to capture gene-gene relationships that are likely to actually exist in the corresponding tissue. However, there is no independent source of data to obtain tissue-specific relationships that are not themselves inferred based on high-through profiling data. Therefore, we decided to create our tissue-aware gold standard by subsetting our naive gold standard with genes known to be expressed in the tissue.*

*Finally, we agree with the reviewer that, even if two genes are expressed, tissue-specific regulation could influence their functional relationship. However, currently tissue-specific regulation can only be inferred from tissue-specific gene expression data (such as the ones used as input in our study), but using this to create the tissue gold standard and evaluate the networks inferred from those very datasets would be circular and, therefore, a poor evaluation. Therefore, we chose to evaluate using our tissue-aware gold standards.*

Comment 1.3

While the authors exhaustively compare the different workflows based on the area under the precision recall curve using the GO annotation based "true" networks, I expected to see additional supporting analyses using other approaches to get at a "true" or expected network. For example, one could use spike-ins or mixture / dilution data, as was previous done for microarray data in:

McCall, Matthew N., and Anthony Almudevar. "Affymetrix GeneChip microarray preprocessing for multivariate analyses." Briefings in bioinformatics 13.5 (2012): 536-546.

*We agree that evaluation using other gold standards is indeed valuable and we spent a considerable amount of effort trying to create such gold standards without much success due to the lack of appropriate external datasets. For example, we attempted to create gold standards based on groups of genes co-bound by the same transcription factor (in ChIP-Seq experiments). However, physical binding of transcription factor does not necessarily indicate functional interaction between the transcription factor and the target gene nor does it indicate co-regulation (coexpression) of the target genes. This limitation was apparent from our observation that coexpression networks evaluated on these TF-binding-based gold standards had random performance at best and worse than random performance otherwise, regardless of workflow, sample size, or data quality.*

*We also attempted to create a gold standard based on groups of genes co-annotated to only tissue-specific GO biological processes. However, there was very little experimental annotation in this data to create gold standards that span tens of thousands of genes for many tissues.*

*We did not attempt to use spike-in data for similar reasons: limited data that prevents conducting an evaluation on a large-enough scale to be comfortable drawing general conclusions. To our knowledge, there is not a large collection of readily-available spike-in data from multiple sources and tissues to leverage for this purpose. RNA-seq experiments are quite sensitive to technical effects when considering final quantification of each gene count in a given sample. These technical effects are a combination of the specific transcripts of interest (GC content, length, reverse transcriptase binding site sequence, etc) and the overall distribution of the population of RNA/cDNA in the sample library (whether rRNA depletion or polyA+ tail selection is used, which tissue the sample comes from, sequencing protocol, etc). All these factors can have significant effects on total read counts and, thus, gene count quantification. And, spike-in controls are not immune to these effects [Qing et al.]. As we discuss throughout the paper, different normalization and network transformation techniques handle these technical biases differently, whether explicitly or implicitly. Therefore, to call a method or workflow “robust”, it must work well over a large number of datasets that encompass datasets with any number of a variety of technical biases. In our study, we check for robustness by using a large number of primary bulk RNA-seq samples (over 15,000) from over 35 tissues and 200 independent studies that were all quantified into gene counts by the same alignment software. It would be a considerable effort to collect data with spike-ins from many sources and process the raw reads into counts data for another large set of data, as we used Recount2 to take away the variability of using different alignment software. Currently, there does not seem to be a single, high-quality, large dataset for spike-in RNA-seq data that would correspond to something like the data used in our GTEx analysis where the raw reads are converted to gene counts using the same quality-control and quantification procedure (like in Recount2) to take away the variability of using different alignment software.*

*Furthermore, even if there is enough spike-in data available, it is not certain that it would be a useful evaluation. The purpose of spike-in experiments is often to estimate the precision and accuracy of the sequencing technology. So, typically, the concentration of the spike-in is equal across samples in a dataset. Even in cases where the concentration of a given spike-in probe is varied across samples, the point of these spike-ins is to find the limit of detection or to be at a detectable level so that they can be used for quantification. This means that, in many cases, we would only be able to evaluate spike-in oligos with a nominal correlation of one or zero (as was done in the McCall and Almudevar paper). To be clear, this means that we would not be able to evaluate any correlation between 0 and 1. This limitation skews the assessment of workflows to an evaluation of genes that are perfectly coexpressed and highly ‘expressed’ in at least some samples. As discussed briefly in the Discussion section of our manuscript, the mean-correlation relationship bias (the observation that highly-expressed genes tend to be more highly-coexpressed in coexpression analysis) might make this type of gold standard rather easy to achieve for all workflows. Such a gold standard does not represent a large number of genes that are never highly expressed but are nonetheless genes of great interest.*

*Qing T, Yu Y, Du T T, et al. mRNA enrichment protocols determine the quantification characteristics of external RNA spike-in controls in RNA-Seq studies. Sci China Life Sci, 2013, 56: 134–142. Link*

Comment 1.4

Minor Essential Revisions

While the results of the study could also be found on the website:

https://urldefense.com/v3/__https://krishnanlab.github.io/RNAseq_coexpression__;!!HXCxUKc!hR3GY_jlT1P_TukGqAYH3sPU9MHkg2U1-2ucOqmbUlmCgolYTf0Q6cRih-rv$

I was not able to find code to reproduce the analyses.

*We thank the reviewer for bringing up this important point. We have now created a GitHub repository with all of the scripts necessary to regenerate our results and for researchers to create coexpression networks from their datasets of interest: https://github.com/krishnanlab/RNAseq_coexpression*

Reviewer 2

========

The paper aims at tackling the relevant and interesting challenge of how to process RNA-seq data with the goal of constructing co-expression networks. This is as of yet unclear, and the paper therefore aims to fill an important gap in the literature. I therefore think the topic itself is interesting, but I feel that the authors have missed several opportunities in this paper that would significantly improve it.

Major comments

Comment 2.1

- In the Background section, the authors discuss the terms ‘normalization’ and ‘transformation’. At first read, I had considered normalization to stand for within- /between-sample normalization such as CPM or TMM, and transformation to stand for transformation of the data, such as log-transformation. It turns out the authors restrict the use of the word ‘transformation’ to transform the obtained co-expression network. The authors should more clearly differentiate between these two terms. A log-transformation of the data can be considered a normalization; these two terms are not necessarily separate ideas (as they seem to be considered in the manuscript) of working with the data and the Background section would benefit by, from the start on, clearly mentioning what the authors mean when they use these words.

*We thank the reviewer for pointing out this confusing language. We have now added text to the Background section to clarify these terms. Briefly, we now refer to data normalization (CPM, TMM, etc.), data transformation (log, asinh, etc.), and network transformation (CLR and wTO).*

*Relevant text:*

*Nevertheless, multiple experimental factors impact the quantification of the expression of individual genes and the coexpression between pairs of genes, making it necessary to normalize and transform high-throughput gene expression data before downstream analysis. For RNA-seq data, examples of factors that affect the number of reads mapped to a gene include gene length, gene sequence, sample RNA population, and sequencing depth. Some factors have a greater effect on comparisons of gene counts within a single sample (‘within-sample’ effects) while others have a greater effect on comparisons of the same gene’s counts in different samples (‘between-sample’ effects) [4]. Many data normalization and transformation techniques have been developed to explicitly address one or more of these factors. An additional adjustment that can be considered particularly in coexpression analysis is network transformation, which is applied after calculating correlation between all gene pairs. Coexpression networks are noisy and can indiscriminately capture indirect interactions due to being estimated from noisy, steady-state gene expression data. Hence, previous studies have proposed methods to modify the raw coexpression network to upweight connections that are more likely to be real and downweight spurious correlations based on the topology of the network [5,6]. Together, appropriately normalizing and transforming RNA-seq data along with adequately transforming the coexpression strengths should yield more accurate estimates of gene-gene coexpression that best capture functional relationships between genes.*

Comment 2.2

- The authors evaluate a range of pre-processing methods to evaluate the estimation of coexpression networks on a number of datasets. However, the authors do not release these obtained results as a resource to the community, which seems to be a missed opportunity. A consensus co-expression network as well as tissue-specific co-expression networks could be constructed and provided as valuable resources.

*We agree with the reviewer that releasing individual or consensus coexpression network collections would be valuable to the community. However, this would be a considerable undertaking (a separate research project) because of the following reason:*

*Several resources for functional interaction networks and coexpression networks already exist (HumanBase, GIANT v2, STRING, BioGRID, CoCoCoNet). These resources, which were created with the purpose of publicly releasing networks, have generally used a more comprehensive set of the available expression data. There are general and tissue-specific gene networks in these resources, and some even contain networks from multiple species created using the same methods so that interspecies comparisons can be made. On the other hand, though we used hundreds of datasets, we chose only a subset of data (available in Recount2) due to a number of practical aspects we needed to consider when performing a benchmarking study (e.g., enough number of samples per dataset, enough number of datasets per tissue, samples from primary tissue sources, etc). The resources mentioned above were also created by making careful choices about how to combine multiple networks into a single consensus network. Currently, it is not clear which of these methods for building a consensus network would be appropriate for combining RNA-seq coexpression networks. Therefore, anything we could currently release would be inferior to these existing resources, and the effort it would take to match these resources in terms of the underlying methods, software infrastructure, and database management is unfortunately outside the scope of the study described in this manuscript. This is a great idea and we will certainly work on this as part of a future study.*

*Nevertheless, since we have now released code to repeat our analysis, any researcher can use our code to recreate these networks on their own.*

Comment 2.3

- The gene filtering step removes genes which have less than one CPM in at least 80% of the samples in at least one dataset. Upon first reading, this looked stringent. Indeed, genes with tissue-specific expression would be removed when using this criterion (as they would not be expressed in a dataset not considering that tissue), significantly reducing the relevance of the tissue-specific co-expression evaluation. However, the authors still retain over 20K genes. Can the authors provide some intuition about this somewhat surprising Result?

*We designed the gene filtering step to, in fact, be very relaxed. First, we keep all the original gene expression datasets separate and do not combine samples from multiple datasets for any analysis. Then, we only remove genes that do have less than 1 CPM in ≥80% of samples in at least one dataset. Conversely, if a gene is expressed (CPM ≥ 1) in at least 20% of the samples in even one of the 256 SRA datasets, we would retain that gene in all our analysis. So, we are only removing genes that effectively unexpressed in all the datasets, thus retaining more than 20K genes.*

Comment 2.4

- Several pre-processing steps must be made more clear / better argumented. At least, the robustness of the results should be assessed by including a ‘none’ option in each step, in particular the steps sample selection, gene filtering, gene type filtering, and hyperbolic arcisne.

*We thank the reviewer for pointing out the need for clarity of our pre-processing steps. We have now added wording to the Methods sections (Preprocessing, Gene type filtering, and Hyperbolic arcsine transformation) that justifies the pre-processing steps to make our procedure clearer and better supported.*

*As for gene and sample filtering, we have adopted very relaxed criteria and completely leaving out these steps would result in retaining completely non-informative data (i.e., genes that are effectively unexpressed in all the datasets or samples that, for technical reasons or otherwise, have very few detected genes). We chose non-stringent criteria so that quality of data did not play a large role in the network evaluation (e.g., genes not being detected from lower sequencing depths etc). As mentioned in the previous response, the relaxed gene filtering step still retains more than 20K genes. All samples that were removed by our sample-filtering step came from datasets that were subsequently removed because those datasets dropped below the 5-samples-per-dataset requirement. So, our sample filtering step was only removing very low-quality datasets and none of the datasets we included in the benchmarking previously contained a sample we filtered out. Thus, sample filtering in itself did not influence any of the data we ultimately used in this study.*

*The gene type filtering step retains only ‘long’ RNA genes. Shorter reads from short genes are more difficult to accurately map and quantify reproducibly through RNA-seq [Garber et al, Łabaj et al]. Besides being less reproducible, there is currently very little in terms of functional information for the short genes we filtered out (mostly small RNA gene types). Therefore, even if we estimate the relationships between the genes that would have been kept in a “none” option for gene type filtering, we do not have a way to evaluate these predictions.*

*The point about the hyperbolic arcsine transformation is an interesting one. A log transformation is standard procedure when working with RNA-seq (or even microarray) data, as the expected variance grows with the mean for gene counts [Love et al.]. Since gene counts for several genes can be zero, the typical manner in which log transformation is applied to RNA-seq data is to add a pseudocount (of 1, for example) to every gene’s count (say, ‘x’) before taking the log (i.e. log(x+1)). However, adding a constant pseudocount to all genes is disadvantageous because low gene counts are disproportionately increased compared to high gene counts before log transformation (e.g. 1 + 1 is a 100% increase for a gene count of 1, but 941 + 1 is almost a negligible increase). Different studies use different constant pseudocounts (1, 0.5, 0.25, etc) for all the genes. The hyperbolic arcsine (asinh) transformation is defined along the entire real number line and circumvents the need for predefining a constant pseudocount and instead calculates a “pseudocount” that is proportional to the original gene count.*

*However, even though log transformations are standard in the literature, asinh has not been explicitly tested to our knowledge. So, we have now compared doing a hyperbolic arcsine transformation vs not doing a transformation at all (and, related to a later point, to the DESeq2 transformations VST and rlog). Our comparison shows that the hyperbolic arcsine transformation is superior in all tested cases when quantifying gene coexpression using the Pearson correlation. (Log or asinh transformation does not impact Spearman correlation calculation because these transformations preserve the ranking of each gene’s expression levels across samples and Spearman correlation works on these ranks rather than actual values.)*

*Garber M, Grabherr MG, Guttman M and Trapnell C. Computational methods for transcriptome annotation and quantification using RNA-seq. Nature Methods 2011. Link*

*Łabaj PP, Leparc GG, Linggi BE, Markillie LM, Wiley HS, and Kreil DP. Characterization and improvement of RNA-Seq precision in quantitative transcript expression profiling. Bioinformatics 2011. Link*

*Love MI, Huber W, and Anders S. Moderated estimation of fold change and dispersion for RNA-seq data with DESeq2. Genome Biology 2014. Link*

Comment 2.5

Some examples on unclarities:

o The authors discuss ‘qauntile normalization’ (QNT). It is unclear whether this refers to ‘full-quantile normalization’ first adopted on RNA-seq data, I believe, by Bolstad et al. (2003), or some other form of quantile normalization.

*We thank the reviewer for catching this. We mistakenly left this reference out. It has now been added to the between-sample normalization section in Methods.*

Comment 2.6

o TMM and UQ normalization produces offsets, which are often incorporated into GLMs for differential expression analysis. How are these offsets used here? Are normalized counts calculated using these offsets? Providing the equations for this seems necessary.

*Thanks for pointing this out. The counts are divided by the calculated offsets. We have added this sentence to the between-normalization section in Methods and implementation details can be found in the scripts we have now released in the GitHub repository: https://github.com/krishnanlab/RNAseq_coexpression*

*Relevant text:*

*“We performed trimmed mean of M-values (TMM) and UQ normalization of the counts data using the edgeR package to calculate the scaling factors, which were then used as a divisor for each gene count in the proper sample.”*

Comment 2.7

o The preprocessing steps are introduced as being popular, but I personally have rarely read papers that use the hyperbolic arcsine transformation, and this does not seem to be a standard choice to me. The authors also do not argument it.

*We agree that we should clarify this point and we have now added a specific section about this transformation to Methods (along with references) and discuss it further in the Discussion section under “Impact of data transformation”.*

*Briefly, we have used the asinh transformation (log( x + √(x2 + 1) )) as a more general case of the commonly-used log transformation of gene counts along with a constant pseudocount (i.e., log( x + 1 )). As mentioned in our response to Comment 2.4 above, a constant pseudocount unevenly impacts genes with low, nominal, and high expression levels whereas the pseudocount in the asinh scales with the gene’s expression level.*

Comment 2.8

- Tissue-specific gold standard networks used for evaluation are constructed by subsetting the global gold standard network only using genes known to be expressed in a particular tissue. Is ‘known to be expressed in a particular tissue’ derived from the data, or a database? Also, this creation of tissue-specific gold standard networks does not seem to account for the fact that genes might be expressed in 2+ tissues, but differentially coexpressed in these different environments, which seems very relevant to evaluate tissuespecific co-expression. Can the authors comment on, and possibly rectify, this?

*The reviewer is bringing up an important point and we have put considerable thought into it before making the choices we made in this study.*

*First, we obtained tissue-specific genes from the TISSUES 2.0 database “knowledge channel” [Palasca et al.]. These are manual annotations of tissue expression of genes/proteins provided by UniProtKB. After we obtain these tissue-gene associations, we do not perform any further filtering based on the number of tissues a given gene is expressed. Therefore, any given gene can indeed be included in multiple tissues if it is associated in the TISSUES 2.0 database with multiple tissues.*

*Second, tissue-specific differential coexpression is indeed an important factor because tissue-specific gene regulation could influence the functional relationship between a pair of genes even if the two genes are expressed in that tissue. However, as noted in response to Reviewer 1’s Comment 1.2, currently tissue-specific differential coexpression can only be inferred from high-throughput tissue-specific gene expression data (such as the ones used as input in our study), but using this data to create the tissue gold standard and evaluate the networks inferred from those very datasets would be circular and, therefore, lead to a poor evaluation..*

*For both these reasons, to be more accurate, we changed mentions of “tissue-specific” to “tissue-aware.” The purpose of this evaluation was to make sure as many networks as possible were also being evaluated on their ability to capture gene-gene relationships that are likely to actually exist within the given tissue. However, with no independent source to obtain tissue-specific relationships, we decided to subset our naive gold standard with genes known to be expressed in the tissue. We think “tissue-aware” more accurately reflects this.*

*We have now added text to the Network Evaluation section of Methods so that it now reads:*

*“We created the tissue-aware gold standards for as many tissues as possible by subsetting the naive gold standard based on genes known to be specifically expressed in a particular tissue. We obtained tissue-aware genes from the TISSUES 2.0 database Knowledge channel [47]. The knowledge channel contains curated manual annotations of tissue expression provided by UniProtKB.”*

*Palasca O, Santos A, Stolte C, Gorodkin J, and Jensen LJ. TISSUES 2.0: an integrative web resource on mammalian tissue expression. Database 2018. Link*

Comment 2.9

- The authors evaluate some between-sample normalization methods, but the number of methods is still limited to three, and a ‘no normalization’ option. It might be useful to consider more options that are commonly used, e.g., DESeq2 normalization.

*We chose methods that we observed in the literature that did not require prior knowledge or specific information about a given dataset. Originally, DESeq2 transformation methods (rlog and VST) were left out because we only saw them used in differential expression analyses or visualization. However, we agree with the reviewer that there is no reason these methods could not be used for coexpression analysis. So, now, we have performed this comparison. We observe that neither VST nor rlog performed particularly well and, further, rlog takes a significant amount of computing time for large datasets. In effect, these findings do not change our recommendations. In light of this point and the few others mentioned above, we have now added a new sub-section “Impact of Data Transformation” to Discussion to present these results and the accompanying figures are now part of the supplement (Fig. S10–13).*

Comment 2.10

- Networks are always constructed based on pairwise Pearson correlation between genes. There is an obvious missed opportunity to include co-expression measures in the evaluation benchmark. It would be a great contribution to study the interaction between pre-processing and evaluation metrics. Indeed, other work such as Teng et al. (2012) have demonstrated the clear disadvantages of using Pearson correlation on heavy-tailed count data such as RNA-seq Datasets.

*Thanks to the reviewer for raising this point. We were unable to find the “Teng et al. (2012)” reference. We were able to locate one other study comparing (only) correlation measures that showed spearman and two other methods as top performers [Kumari et al.]. However, the purpose of our study was to evaluate data normalization/transformation and network transformation methods (and their combinations) because while there are several often-used methods for data normalization and, to a lesser extent network transformation in the literature over the past decade or so, only Spearman and Pearson correlations have been commonly used for building coexpression networks.*

*Therefore, to address this point directly, we ran the top ten workflows using Spearman correlation in place of Pearson correlation and compared the results. The top workflows we selected were workflows that do not include RPKM, because TPM and CPM (the other within-sample normalization methods) clearly outperform RPKM. These are also workflows that do not include wTO or quantile normalization because both methods decrease performance in all combinations they were evaluated in. In almost all cases, Pearson correlation is the better measure, especially for SRA datasets (i.e. datasets typically generated by individual research labs) and particularly in ensuring the accuracy of the top-scoring edges. For this reason, we only use Pearson correlation in our study. In theory, it is possible to evaluate additional less-frequently-used correlation measures against all workflows but that would significantly increase the amount of computation time and is outside the scope of this work. Specifically, setting aside the GTEx resampling analysis, adding rlog, VST, and their combinations with network transformation methods brings us to a total of 10,332 genome-scale networks. This change would increase the GTEx resampling analysis from 72,900 networks to 87,480 networks. Each additional correlation measure evaluated, therefore, requires another 97,812 networks to be built and evaluated. Hence, given our goal is to systematically explore the impact of normalization and transformation methods, testing these with the best and most-frequently-used correlation measures would be sufficient.*

*Kumari S, Nie J, Chen H, Ma H, Stewart R, Li X, Lu M, Taylor WM, Wei H. Evaluation of Gene Association Methods for Coexpression Network Construction and Biological Knowledge Discovery. PLOS ONE 2012. Link*

*van Dam S, Võsa U, van der Graaf A, Franke L, de Magalhães JP. Gene co-expression analysis for functional classification and gene–disease predictions. Briefings in Bioinformatics 2018. Link*

Comment 2.11

- The authors did not provide code to reproduce the results in the manuscript. Please deposit your code in a relevant repository such that they may be browsed and re-used by other Scientists.

*We thank the reviewer for bringing up this important point. We have now created a GitHub repository with all of the scripts necessary to regenerate our results and for researchers to create coexpression networks from their datasets of interest:* [*https://github.com/krishnanlab/RNAseq_coexpression*](https://github.com/krishnanlab/RNAseq_coexpression)

Comment 2.12

- Genes in the same GOBP term are positively co-expressed. However, asides from two exceptions relating to sets of genes that are co-expressed with many of the same genes or coannotated in general GOBP terms, I interpret the writing that all other genes are decided upon to be negatively co-expressed. This seems like quite the stretch, as the authors seem to consider two independent genes to be negatively co-expressed, which can have drastic consequences to the evaluation procedure.

*As the reviewer suspects, creating the gene functional relationship gold standard from GOBP is not straightforward and we have put a lot of care into this procedure. First, we do not use all GOBP terms to construct our gold standard. Following previous work [Greene et al.], we use only 607 “specific” GOBP terms manually selected by a team of seven graduate students and postdocs (with training in cell/molecular biology and genetics) for being experimentally testable. Only gene pairs co-annotated to any of these specific terms – based on experimental evidence – are considered to have a positive relationship in our gold standard.*

*Similarly, as the reviewer points out, assuming every other gene pair is a negative (not functionally related) would indeed be far too strong an assumption. So, the team also selected a set of 75 “intermediate” GOBP terms such as “protein folding” or “cell proliferation”. Then, to be considered as a negative relationship in the gold standard, gene pairs must meet the following three criteria:*

*1. The two genes are not co-annotated to any intermediate term*

*2. The two genes are not co-annotated to significantly overlapping specific terms (hypergeometric test; p-value <0.05)*

*3. Each gene individually has at least one annotation to a specific term*

*Gene pairs that are co-annotated to intermediate terms (criterion 1) would be considered too close in general function to be sure that they are not functionally related. The hypergeometric test (criterion 2) prevents gene pairs that may share a function (due to being annotated to two overlapping terms) from being labeled as negatives. Requiring a specific term annotation for each gene ensures that no assumptions are made about genes that have not been experimentally studied before.*

*Greene CS, Krishnan A, Wong AK, Ricciotti E, Zelaya RA, Himmelstein DS, Zhang R, Hartmann BM, Zaslavsky E, Sealfon SC, Chasman DI, FitzGerald GA, Dolinski K, Grosser T and Troyanskaya OG. Understanding multicellular function and disease with human tissue-specific networks. Nature Genetics 2015. Link*

Comment 2.13

- It is unclear to me what the authors consider as a ‘true positive’, ‘false positive’ etc. Do the authors consider a true positive as soon as the sign of the Pearson correlation coefficient matches the sign of the edge in the gold standard? Is significance considered? Multiple testing?

*The gold standard contains thousands of gene pairs that either have a functional relationship (positive) or do not have a functional relationship (negative), defined based on experimental gene co-annotations to specific GOBP terms (see above). Then, we evaluate each coexpression network (derived from a single RNA-seq dataset analyzed using any one of the workflows) by comparing it to this gold standard by essentially asking the following question: do gene pairs that have very high coexpression strengths (i.e. high correlation coefficients) tend be functionally related to each other based on the gold standard?*

*We answer this question quantitatively by calculating the area under the precision-recall curve for that coexpression network in the following manner:*

*- We rank all the gene pairs in the network from highest to lowest correlation.*

*- Then, at various cutoffs of correlation strength from high to low, we calculate the number of true positives, false positives, true negatives, and false negatives.*

*- - Gene pairs with a correlation value above the cutoff and,*

*- - - Functionally related in the gold standard (i.e., positive) are ‘true positives’ (TP).*

*- - - Not functionally related in the gold standard (i.e., negative) are ‘false positives’ (FP).*

*- - Gene pairs with a correlation value below the cutoff and,*

*- - - Functionally related in the gold standard (i.e., positive) are ‘false negatives’ (FN).*

*- - - Not functionally related in the gold standard (i.e., negative) are ‘true negatives’ (TN).*

*- - These TP, FP, FN, and TN values are combined to calculate the precision (= TP / ( TP + FP ) ) and recall (= TP / ( TP + FN ) ) at that cutoff.*

*- Finally, all the precision and recall values at the various correlation cutoffs are used together to build the precision-recall curve and the area under this curve is used to quantify the ability of the coexpression network to recapitulate gene functional relationships in the gold standard.*

*Significance and multiple hypothesis testing are not considered for building precision-recall curves or ROC curves because there is no hypothesis testing.*

Comment 2.14

General comments

- No preprocessing at all, neither on the data nor the co-expression network, performs best in the SRA evaluation. This is pretty surprising, and should be discussed and explored. It is also the sixth best method in the GTEx evaluation, and only performing CLR network transformation is the fourth best method, which is similarly pretty surprising. Even in datasets with a high read count diversity (Figure 5c,g), no between-sample normalization performs very well for GTEx and best for the SRA evaluation.

*The workflow being described here (referred to as the Counts workflow in the manuscript) does indeed not include any normalization but does include the asinh transformation, which stabilizes the variance. Despite its good performance, we still recommend normalization. This is previously included in our recommendations: “Even though no normalization (Counts) leads to good performance in our study, applying the additional normalization step is prudent to ensure robustness against variabilities specific to a new dataset.”*

*Nevertheless, in the new “Impact of data transformation” sub-section under Discussion, we have now added a note to briefly discuss the surprisingly good performance of the Counts workflow.*

*Relevant text:*

*It is worth noting that the Counts workflow performs well despite not incorporating any within- or between-sample normalization but only an asinh transformation. We speculate that this good performance is due to the variance stabilization provided by the asinh transformation along with the across-sample normalization of gene expression vectors inherent within the calculation of the Pearson correlation coefficient.*

Comment 2.15

Minor comments

- Table 1 would benefit from a ‘Number of experiments/datasets’ row.

*This is a good suggestion. We have now added this to Table 1.*

Comment 2.16

- The authors discuss the preprocessing procedure ‘counts’ which I assume is no withinsample normalization, no between-sample normalization and no network transformation, but this isn’t defined in the manuscript.

*Thanks for pointing this out. We have now added the following to the Results section where we define workflows and methods: “The Counts workflow uses no within-sample normalization, between-sample normalization, or network transformation, but is still transformed with the hyperbolic arcsine function.”*

Comment 2.17

- In the Methods: Network evaluation section, the authors write that they “assembled these gold standards by beginning with a set of manually-selected GOBP terms”. Some intuition on the previous work in [39] as to how/why these were selected would be useful.

*Thanks for the suggestion. We have now added some text to the Network evaluation in Methods so that it now reads:*

*“We assembled these gold standards by beginning with a set of manually-selected Gene Ontology Biological Process (GOBP) terms [42] that were deemed to be specific enough to be confident that any gene annotated to them could be verified via experimental follow-up. Specifically, curators were considering the question “if unknown gene/protein G were predicted to be annotated to GOBP term T, would that be enough to consider experimentally testing this relationship between G and T?” Then, any pair of genes that were co-annotated to the same specific GOBP term was set as a positive edge in the gold standard. We only used annotations based on experimental (GO evidence codes: EXP, IDA, IPI, IMP, IGI, TAS) or curated evidence (IC). We explicitly ignored gene-term annotations made based on expression (GO evidence code: IEP) to avoid circularity when comparing coexpression-derived interactions to this gold-standard. We next had to determine which pairs of genes among the ones with at least one positive edge could be declared as negative edges. Following previous work, we ignored gene pairs not co-annotated to any specific term but still co-annotated to two terms that are similar to each other (determined based on the overlap between sets of genes annotated to the two terms; hypergeometric test; p-value <0.05). We also ignored gene pairs that were not co-annotated to any specific term but were co-annotated to certain intermediate GOBP terms, thus introducing ambiguity in whether they are functionally related or not. All remaining gene pairs were considered negatives.”*

*We have also added a new Supplementary Note that covers our rationale for building this gold standard and the other gold standards we considered.*

Comment 2.18

- The authors write that they got the tissue-specific genes from the TISSUES 2.0 database,

and mention a z-score and Knowledge channel. It is unclear what is meant with this.

*We agree that the text in that section was unclear and we have now edited to:*

*“We created the tissue-aware gold standards for as many tissues as possible by subsetting the naive gold standard based on genes known to be specifically expressed in a particular tissue. We obtained tissue-aware genes from the TISSUES 2.0 database Knowledge channel [47]. The “knowledge channel” contains curated manual annotations of tissue expression provided by UniProtKB.”*

Comment 2.19

- It is not mentioned how ‘sample similarity’ and ‘read count diversity’ are calculated and

therefore the labels on the x-axis of Fig5 and related figures cannot be interpreted.

*We have now added a note “(see Experimental factor analysis in Methods)” to this Results section of the paper to make it more clear that this is explained elsewhere.*

*The Experimental factor analysis sections of Methods (provided below) contains information about how ‘sample similarity’ and ‘read count diversity’ are calculated:*

*“We define sample similarity for a given dataset as the median spearman correlation between all samples using the 50% most variable genes in the GTEx tissue they came from for the resampled GTEx datasets, or the median spearman correlation between all samples using the 50% most variable genes in each individual dataset in the case of the SRA networks. Read-count diversity is calculated by summing the gene counts of each sample in a given dataset and taking their standard deviation.”*

Comment 2.20

- Genes that are annotated in the same GOBP term are considered to be positively coexpressed, which seems reasonable. How are genes that are present in multiple GOBP

terms handled?

*Each gene would have functional edges with all their co-annotated genes in each manually-selected GOBP in our gold standard. As an example, say, specific term GOBP 1 has genes A, B, and C, and specific term GOBP 2 has genes A, D, and E. The genes with true positive relationships would then be A-B, A-C, B-C, A-D, A-E, and E-D.*

**Second round of review**

**Reviewer 1**

I appreciate the thoughtful responses to my previous comments provided by the authors; however, I still believe that the focus on GO annotation (no matter how carefully done) severely limits the interpretation and reliability of the results. While other benchmark data sets and corresponding assessments may have their own limitations, by examining the performance in different contexts, the authors will be able to provide a more definitive overall assessment of performance.

**Reviewer 2**

The authors have made substantial revisions to their work, which are greatly appreciated. However some major points remain.

·	Comment 2.2.: The authors make a point that it could be a significant undertaking to release the co-expression networks that they have generated, in particular, to assess how one could best construct a consensus network. I still believe that it is unfortunate that the results or not provided - at least in summarized form - even though the code is. Would a simple alternative work, e.g., providing a global consensus network where for each pair of genes the median Pearson correlation is provided using the best workflow?

·	Comment 2.3: I thank the authors for carefully exploring the hyperbolic arcsine and other transformations; the additional Supplementary Figures are very useful.

·	Comment 2.6: I am worried about the use of the TMM and upper-quartile (UQ) normalization factors. In par¬ticular, the use of normalization factors directly to get normalized counts is inappropriate (as done here https: //github.com/krishnanlab/RNAseq_coexpression/blob/main/src/TMM_normalize.R#L33). Indeed, the normalization factors serve to correct the library sizes, producing ‘effective library sizes’, which can then be used to derive normalized counts. This is in contrast to the normalization used by DESeq2 where, in fact, it is appropriate to derive normalized counts by dividing the count by the size factor, since the size factor also incorporates library size effects. The authors should think about calculating normalized counts more carefully.

·	Comment 2.8: Given that the tissue-aware analysis is merely using a subset of the global ground truth network, I am not sure if it still contributes signficantly to the paper. In fact, in the paper, the analysis is introduced as ‘Further, gene activities and interactions vary drastically depending on cell type or tissue. Hence, we created tissue-aware gold standards ...’. However, the ground truth does not allow these interactions to vary depending on cell type or tissue. In fact, because of this, I am not sure if the tissue-aware analysis is significantly contributing to the findings of the paper, as it is an identical assessment restricted to a subset of expressed genes. I would also not use the term ‘specifically expressed’ on line 26 of page 22, and replace it by simply ‘expressed’.

·	Comment 2.10: I apologize for not completing the bibliographic details for the Pearson correlation statement. The paper I referred should have been Teng et al. [2016], which is now also shown in full in the References. I cannot fully agree with the authors’ responses on the Pearson correlation comment. They respond that ‘How¬ever, the purpose of our study was to evaluate data normalization/transformation and network transformation methods (and their combinations) because while there are several often-used methods for data normalization and, to a lesser extent network transformation in the literature over the past decade or so, only Spearman and Pearson correlations have been commonly used for building coexpression networks.’.

Indeed, exactly because of this point, the conclusions made in the manuscript only apply to constructing co-expression networks using Pearson and Spearman correlations as the association metric, and they do not hold globally, or at least we don’t know this based on the results. The optimal preprocessing likely depends on the method that will be used downstream to calculate co-expression. The additional evaluations show that Pearson correlation is a better metric than Spearman correlation, which is fine, but the point here is that the order of optimal preprocessing pipelines can depend on the metric used. While it is true that a large body of metrics use Pearson or Spearman correlation, many methods also use other metrics, see for example methods discussed in Song et al. [2012], but also methods building on machine learning tools, e.g., GENIE3 [Huynh-Thu et al., 2010], which has the goal of constructing regulatory networks but starts with a co-expression network. It should therefore be made clear that these results only hold when using Pearson and Spearman correlation.

·	Comment 2.12 and 2.13: I thank the authors for detailing the evaluation procedure. I would suggest to talk about ‘independent genes’ rather than ‘a gene pair is a negative’, as the latter could refer to genes being anti-correlated (negatively co-expressed). It is now clear that the authors are not benchmarking co-expression in terms of anticorrelation, i.e., the authors ignore the existence of anticorrelation, and this should be mentioned in the paper.

References

Michael I Love, Wolfgang Huber, and Simon Anders. Moderated estimation of fold change and dispersion for RNA-seq data with DESeq2. Genome Biology, 15(12):550, dec 2014. ISSN 1465-6906. doi: 10.1186/s13059-014-0550-8. URL http://genomebiology.com/2014/15/12/550.

Davis J McCarthy, Yunshun Chen, and Gordon K Smyth. Differential expression analysis of multifactor RNA-Seq experiments with respect to biological variation. Nucleic acids research, 40(10):4288–97, may 2012. ISSN 1362-4962. doi: 10.1093/nar/gks042. URL http://www.pubmedcentral.nih.gov/articlerender.fcgi?artid= 3378882{\&}tool=pmcentrez{\&}rendertype=abstract.

Mingxiang Teng, Michael I Love, Carrie A Davis, Sarah Djebali, Alexander Dobin, Brenton R Graveley, Sheng Li, Christopher E Mason, Sara Olson, Dmitri Pervouchine, Cricket A Sloan, Xintao Wei, Lijun Zhan, and Rafael A Irizarry. A benchmark for RNA-seq quantification pipelines. Genome Biol., 17:74, apr 2016.

Lin Song, Peter Langfelder, and Steve Horvath. Comparison of co-expression measures: mutual informa¬tion, correlation, and model based indices. BMC Bioinformatics, 13(1):328, dec 2012. ISSN 1471-2105. doi: 10.1186/1471-2105-13-328. URL http://bmcbioinformatics.biomedcentral.com/articles/10.1186/ 1471-2105-13-328.

Vaˆn Anh Huynh-Thu, Alexandre Irrthum, Louis Wehenkel, and Pierre Geurts. Inferring Regulatory Networks from Expression Data Using Tree-Based Methods. PLoS ONE, 5(9):e12776, sep 2010. ISSN 1932-6203. doi: 10.1371/journal.pone.0012776. URL https://dx.plos.org/10.1371/journal.pone.0012776.

**Authors Response**

**Point-by-point responses to the reviewers’ comments:**

Reviewer 2

=======

The authors have made substantial revisions to their work, which are greatly appreciated. However some major points remain.

Comment 2.2

The authors make a point that it could be a significant undertaking to release the co-expression networks that they have generated, in particular, to assess how one could best construct a consensus network. I still believe that it is unfortunate that the results or not provided - at least in summarized form - even though the code is. Would a simple alternative work, e.g., providing a global consensus network where for each pair of genes the median Pearson correlation is provided using the best workflow?

*We agree with the potential utility of these networks generated using our best workflow. Therefore, we have now invested considerable effort in releasing all the individual networks. The networks are now in a publicly-accessible google drive folder but are being uploaded to Zenodo, a public data repository zenodo that will create a unique data record and an associated DOI (see details below). We are working with Zenodo to perform this upload, which will be completed within the next week, but didn’t wish to delay this resubmission any further.*

*Current (temporary) data location: https://drive.google.com/drive/folders/1Ns4FvpFpmPiceLDjWlj9lX3fTKQonA8h?usp=sharing.*

*Zenodo data record where the data will reside very soon: https://zenodo.org/record/5510567.*

*Dataset DOI: https://doi.org/10.5281/zenodo.5510567.*

Comment 2.3

I thank the authors for carefully exploring the hyperbolic arcsine and other transformations; the additional Supplementary Figures are very useful.

*Thank you.*

Comment 2.6

I am worried about the use of the TMM and upper-quartile (UQ) normalization factors. In par- ticular, the use of normalization factors directly to get normalized counts is inappropriate (as done here https://github.com/krishnanlab/RNAseq_coexpression/blob/main/src/TMM_normalize.R#L33

Indeed, the normalization factors serve to correct the library sizes, producing ‘effective library sizes’, which can then be used to derive normalized counts. This is in contrast to the normalization used by DESeq2 where, in fact, it is appropriate to derive normalized counts by dividing the count by the size factor, since the size factor also incorporates library size effects. The authors should think about calculating normalized counts more carefully.

*We first want to thank the reviewer for not just reading our manuscript but taking a look at our code. The fact that this point was brought up speaks to the value of openly releasing the underlying code, which was a lot of hard work from the primary author.*

*We went back and carefully thought about this point and we agree with the reviewer that how we use the TMM and UQ factors is, in fact, important to consider and describe carefully. Therefore, we went back and did two things:*

*Re-calculated TMM and UQ – as the reviewer has pointed out – by using the normalization factors to correct the library sizes.*

*Retained our calculations – i.e., using the normalization factors to adjust the counts without any further division by the library sizes – but gave these calculations new names: Counts adjusted by TMM factors (CTF) and Counts adjusted by UQ factors (CUF).*

*Then, we included all the measures back into our study and tested all workflows using our systematic benchmarking procedure.*

*Including the two new between-sample normalization methods (CUF and CTF) increased the total number of workflows we tested from 30 to 36. See new Figure 1 (included here). After this addition, we have regenerated all our results (including all main and supplementary figures and tables). We have added a new analysis (Figure 6 and S8) that shows the same performance trends of our top workflows when applied to SRA RNA-seq data from a different database (refine.bio) that uses entirely different methods for read alignment and expression quantification. Based on all our results, we now report that CTF and CUF are significantly better between-sample normalization methods and workflows that include them result in significantly more accurate coexpression networks (based on Pearson correlation) from small (SRA) and, especially, large (GTEx) RNA-seq datasets. We have added text across the manuscript – Introduction, Results, Discussion, and Methods – to reflect these changes, including potential reasons for these observations.*

Comment 2.8

Given that the tissue-aware analysis is merely using a subset of the global ground truth network, I am not sure if it still contributes significantly to the paper. In fact, in the paper, the analysis is introduced as ‘Further, gene activities and interactions vary drastically depending on cell type or tissue. Hence, we created tissue-aware gold standards ...’. However, the ground truth does not allow these interactions to vary depending on cell type or tissue. In fact, because of this, I am not sure if the tissue-aware analysis is significantly contributing to the findings of the paper, as it is an identical assessment restricted to a subset of expressed genes.

*This is a good point, which we have thought a lot about and considered very carefully.*

*Due to the lack of interaction-level experimental data for each tissue, gene interactions not present in the global (non-specific) ground-truth network but occur only in a particular tissue will be missed. However, it is still worthwhile asking if, among the interactions that are present in the global ground-truth network, the ones that occur only in a particular tissue are captured by the coexpression network constructed based on data from that tissue. There are two reasons for conducting such a tissue-aware evaluation:*

*To make sure that the coexpression network from a tissue dataset does not end up with a high performance measure (e.g. auPRC) only because it faithfully captures global, ubiquitous gene relationships (e.g., interactions between ribosomal genes or between central metabolism genes) while not capturing interactions involving tissue-specific genes.*

*Consider a coexpression network from a given tissue, say ‘heart’. We should not penalize this network for not capturing relationships between genes that are not expressed in heart (i.e., those specifically expressed in other unrelated tissues). This penalty will manifest in the form of a large number of false negatives because the heart coexpression network will have low coexpression scores for genes unlikely to be functionally related in the heart even though they are “positive” edges in the tissue-naive global gold-standard.*

*To directly address the concern that the tissue-aware evaluation might be “an identical assessment restricted to a subset of expressed genes”, we compared the tissue-aware gold standards between tissues at the level of included genes and included edges (interactions).*

*The heatmaps above (included in the response PDF) – which have now been included in the Supplement as Figure S9 – show the overlap between every pair of tissue-aware gold-standards. Each cell represents the number of (a) genes or (b) edges that are shared between two given tissue-aware gold standards divided by the total number of genes or edges in the smaller of the two tissue-aware gold standards. These heatmaps show that, while each of the tissue-aware gold-standards is composed of a subset of the global gold-standard, they are very different from each other in a meaningful way, i.e., gold-standard of biologically similar tissues are similar to each other at the edge level.*

Comment 2.8.2

I would also not use the term ‘specifically expressed’ on line 26 of page 22, and replace it by simply ‘expressed’.

*Each tissue gene set includes only genes ‘specifically expressed’ in that tissue and do not include all genes generally expressed in that tissue. We particularly collected such genes from the TISSUES database, which includes genes that are preferentially associated with various human tissues based on experimental data. So, by design, these tissue gene sets have very little overlap with each other, as can be seen in panel a of the heatmap above. Therefore, we have retained the term ‘specifically expressed’.*

Comment 2.10

I apologize for not completing the bibliographic details for the Pearson correlation statement. The paper I referred should have been Teng et al. [2016], which is now also shown in full in the References. I cannot fully agree with the authors’ responses on the Pearson correlation comment. They respond that ‘However, the purpose of our study was to evaluate data normalization/transformation and network transformation methods (and their combinations) because while there are several often-used methods for data normalization and, to a lesser extent network transformation in the literature over the past decade or so, only Spearman and Pearson correlations have been commonly used for building coexpression networks.’.

Indeed, exactly because of this point, the conclusions made in the manuscript only apply to constructing co-expression networks using Pearson and Spearman correlations as the association metric, and they do not hold globally, or at least we don’t know this based on the results. The optimal preprocessing likely depends on the method that will be used downstream to calculate co-expression. The additional evaluations show that Pearson correlation is a better metric than Spearman correlation, which is fine, but the point here is that the order of optimal preprocessing pipelines can depend on the metric used. While it is true that a large body of metrics use Pearson or Spearman correlation, many methods also use other metrics, see for example methods discussed in Song et al. [2012], but also methods building on machine learning tools, e.g., GENIE3 [Huynh-Thu et al., 2010], which has the goal of constructing regulatory networks but starts with a co-expression network. It should therefore be made clear that these results only hold when using Pearson and Spearman correlation.

References

Michael I Love, Wolfgang Huber, and Simon Anders. Moderated estimation of fold change and dispersion for RNA- seq data with DESeq2. Genome Biology, 15(12):550, dec 2014. ISSN 1465-6906. doi: 10.1186/s13059-014-0550-8. URL http://genomebiology.com/2014/15/12/550.

Davis J McCarthy, Yunshun Chen, and Gordon K Smyth. Differential expression analysis of multifactor RNA- Seq experiments with respect to biological variation. Nucleic acids research, 40(10):4288–97, may 2012. ISSN 1362-4962. doi: 10.1093/nar/gks042. URL http://www.pubmedcentral.nih.gov/articlerender.fcgi?artid= 3378882{\&}tool=pmcentrez{\&}rendertype=abstract.

Mingxiang Teng, Michael I Love, Carrie A Davis, Sarah Djebali, Alexander Dobin, Brenton R Graveley, Sheng Li, Christopher E Mason, Sara Olson, Dmitri Pervouchine, Cricket A Sloan, Xintao Wei, Lijun Zhan, and Rafael A Irizarry. A benchmark for RNA-seq quantification pipelines. Genome Biol., 17:74, apr 2016.

Lin Song, Peter Langfelder, and Steve Horvath. Comparison of co-expression measures: mutual informa- tion, correlation, and model based indices. BMC Bioinformatics, 13(1):328, dec 2012. ISSN 1471-2105. doi: 10.1186/1471-2105-13-328. URL http://bmcbioinformatics.biomedcentral.com/articles/10.1186/ 1471-2105-13-328.

Vˆan Anh Huynh-Thu, Alexandre Irrthum, Louis Wehenkel, and Pierre Geurts. Inferring Regulatory Networks from Expression Data Using Tree-Based Methods. PLoS ONE, 5(9):e12776, sep 2010. ISSN 1932-6203. doi: 10.1371/journal.pone.0012776. URL https://dx.plos.org/10.1371/journal.pone.0012776.

*Thank you for elaborating this point and we agree. Therefore, now, in the “Recommendations for building coexpression networks from RNA-seq data” section, we have added this phrase “using Pearson or Spearman correlation” to make it clear that our recommendations are applicable for only the most popular expression similarity metrics.*

Comment 2.12 and 2.13

I thank the authors for detailing the evaluation procedure. I would suggest to talk about ‘independent genes’ rather than ‘a gene pair is a negative’, as the latter could refer to genes being anti- correlated (negatively co-expressed). It is now clear that the authors are not benchmarking co-expression in terms of anticorrelation, i.e., the authors ignore the existence of anticorrelation, and this should be mentioned in the paper.

*We certainly appreciate this point and it would indeed be better to make this as clear as possible especially to non-computational scientists who read this paper. Therefore, we have made the following additions to the “Results” and “Methods” (Network evaluation) sections.*

*Results:*

*In the gold standard, this imbalance is reflected by the number of non-interactions (referred to as ‘negative examples’ or, simply, ‘negatives’) far outnumbering the interactions (referred to as ‘positive examples’ or, simply, ‘positives’).*

*Methods – Network evaluation:*

*Then, any pair of genes that were co-annotated to the same specific GOBP term was set as a positive edge in the gold standard that corresponds to a known functional relationship.*

*We next had to determine which pairs of genes among the ones with at least one positive edge could be declared as negative edges, i.e., gene pairs that are unlikely to be functionally related based on prior knowledge. To be clear, ‘positive’ and ‘negative’ are used here based on machine learning parlance to indicate interactions and non-interactions, respectively, and do not correspond to the sign of the relationship. This way, the terms are consistent with how we refer to true/false positive/negative edges.*

**Third round of review**

**Reviewer 2**

The authors have done a good job clarifying remaining comments.
